# Abstracts of papers presented at the 2002 Pittsburgh Conference

**DOI:** 10.1155/S1463924602000196

**Published:** 2002


Journal of Automated Methods & Management in Chemistry
Vol. 24, No. 6 (November–December 2002) pp. 171–208

Taylor & Francis

Taylor & Francis Group

### Abstracts of papers presented at the 2002 Pittsburgh Conference

The following 125 abstracts form Part B of two issues of *Journal of Automated Methods & Management in Chemistry* devoted to abstracts of papers and posters presented at the Pittsburgh Conference held from 17 to 22 March 2002 in New Orleans, LA, USA. The papers and posters covered a range of topics and techniques, each of which provided valuable information to the conferees and exhibitors alike. The attendance figures remained disappointing, but Pittcon is still one of my favourite shows and has done a great job for Analytical Chemistry as a whole. People are tiring of New Orleans as a venue and it has become little more than a trade show. Hopefully, it will revive itself when the next show will be held in Orlando, FL, from 9 to 14 March 2003. Perhaps the attendees will spare some time from Disney and Sea World to visit us at the exhibition and conference.

If you need further information about any of these abstracts, please contact the authors. It is my intention to publish full papers corresponding to some of these abstracts in future issues of the journal.

### Strategies for successful global laboratory information management systems implementations

*Stuart M. Miller, Taratec Development Corporation, LIMS & Lab Automation Solutions, 1170 US Highway 22, Ste 302, Bridgewater, NJ 08807-2933, USA*

Pharmaceutical and life sciences companies world-wide are under enormous pressure to transform their drug discovery and development processes in order to make decisions on whether to fail or promote compound development with more confidence and in a much shorter period of time. Computing and software are playing a vital role in this transformation and as a first step, many life sciences companies are establishing foundations of globally harmonized information systems that are intimately tied to the key business processes. LIMS are one of the key systems that provide much of the data required to make the go/no go decisions, and are often one of the first areas of focus for these global harmonization initiatives. Successfully completing a truly global LIMS project involves the usual array of technical challenges but the regulated environments of the life sciences industry and the diverse make up of the global teams present additional challenges. Simultaneously satisfying FDA and international regulatory requirements with a unified global system and establishing common goals and harmonization of the underlying business processes which meet the requirements of the local sites are typically two of the greatest impediments to the success of these projects. While life sciences companies are eager to reap the benefits of a truly global LIMS, special care must be taken to ensure the global teams are prepared to handle these and other challenges that often put the success of these critical projects at risk of producing merely another set of regionally diverse business processes and information systems. In this presentation, we will recommend proven strategies to adopt and pitfalls to avoid in the planning and execution of a global LIMS implementation in the evolving life sciences enterprise. Contrasting anonymous case studies will be used to illustrate the dos and don’ts of this process.

### An LIMS to support recent developments in stability testing

*Nick Townsend and Martin Waugh, Autoscribe, Ltd, 81 Technology Park Drvie, East Falmouth, MA 02536, USA*

Stability testing is becoming more important in gaining regulatory approval to market new formulations of existing drugs and new drug products. Some companies have begun testing the stability of all drug intermediaries as well as the raw materials and the final packaged product. With the acceptance of standardized storage conditions of temperature and humidity, by several international regulatory agencies, there is a need to replicate commonly used protocols in new studies. Furthermore, many protocols now include ‘condition cycling’ as part of the study, so as to simulate real-life situations better. These new requirements have been accompanied by new regulatory guidelines for electronic record-keeping (21 CFR Part 11) We will present information on a new LIMS that has been designed in cooperation with several pharmaceutical companies to meet these new requirements. It uses an intuitive graphical interface to design and approve new protocols and studies. Container/closure, condition and pull libraries are used, as are study and batch copy functions, to speed the set up and approval of new batches. The system manages container inventories, including a provision to track spare containers and scrap any remaining containers when a study is completed. Examples of screens and reports will be provided along with a full description of how the system maintains an audit trail of all activities.

### Delivering laboratory information in an Internet society

*Mark E. Fish[1] and Hemal Rajani[2], [1]Thermo LabSystems, 1 St George’s Court, Altrincham WA14 5TP, UK, [2]Thermo LabSystems, Analytical Chemistry, Caixa Postal 6154, Campinas, Sao Paulo 13081-970, Brazil*

The emergence of the Internet has been the most significant computing development in recent times, if not ever. This has caused an explosion in the amount of information available on a global scale. The transfer of information and data is a fundamental aspect of analytical sciences. Making this information easily available is an essential requirement from bench to board level and can offer significant competitive advantages in an increasingly competitive market place. An intranet today can be used to create new interfaces for complex decision support systems, drawing from a pool of many previously unconnected databases. In the world of laboratory automation, as with any type of industry, the opportunity of enhanced productivity by improving the flow of information and the development of cost-effective solutions for sharing company information are welcomed with open arms. This paper will examine tools and technological available for companies to deliver laboratory information in both Intranet and Internet environments. The paper will also discuss the benefits and potential pitfalls of delivering laboratory information in an Internet society.

### Electronic laboratory notebooks and electronic record-keeping systems

*Kevin Smith, Thermo LabSystems, R&D Department, 1 St George's Court, Hanover B, Altrincham WA14 5TP, UK*

For hundreds of years scientists have relied on their paper notebooks as a medium for recording their work. Many have dreamed of a paperless solution, but the market for Electronic Lab Notebooks remains in its infancy. It is Thermo LabSystems' view that the technology is now available to enable development of a comprehensive Electronic Lab Notebook and as a result we have been performing some market research involving end users, other vendors, and industry groups such as CENSA (http://www.censa.org) We have categorized the requirements into three broad areas:

- Ease and convenience of use, including mobility, compound document creation, instrument integration, etc.
- Record keeping: addressing regulatory (21 CFR Part 11) and IP issues arising from the long-term storage of records in electronic format.
- Knowledge management: allowing scientists to collaborate through publishing, sharing, and mining the above records, again over the long-term.

The first set of these requirements is relatively easy to meet and there are many current examples (web tablets, word processing packages, LIMS, etc.). However, long-term record keeping and knowledge management require data to be stored in a back end system using 'platform neutral' formats, allowing data to be read after the original application is no longer available. For human readable data, such as text and diagrams, Adobe PDF is a widely accepted format, expected to be readable for many years into the future. For analytical data generated by instruments such as GC/LC, MS, FT-IR, UV, NMR, etc., eXtensible Markup Language (XML) is more suited and again is likely to be readable in many years to come. Based on the above, we are currently working on an Electronic Record Management (ERM) system which we believe will be suitable (1) as a backend for an ELN and (2) as a standalone system for long-term laboratory data archival. The presentation will describe the above issues and possible solutions in more detail.

### Chemometric data evaluation for improved seawater monitoring of chlorinated hydrocarbons by mid-infrared evanescent wave sensors

*Frank Vogt[1], Manfred Karlowatz[1], Lubos Hvozdara[1], Boris Mizaikoff[1] and Martin Kraft[2], [1]Georgia Institute of Technology, School of Chemistry and Biochemistry, 770 State Street NW, Atlanta, GA 30332-0400, USA, [2]Vienna University of Technology, Institute of Analytical Chemistry, Getreidemarkt 9/151, Vienna A-1060, Austria*

The increasing pollution of the world water resources and the marine ecosystems demands for novel on-line and *in-situ* measurement techniques. Scientific goal is to enable concentration surveillance and enhanced understanding of pollutants transport mechanisms. Based on a Bruker Vector 22 mid-infrared FT-IR spectrometer, a novel sensing device is developed for pollutant detection in seawater. This device is designed for submarine measurement tasks by installing a FT-IR spectrometer in a deep-sea housing, which is connected to a polymer coated silver-halide waveguide sensor. This sensor uses the principle of mid-infrared evanescent wave sensing. At the time, the target analytes dissolved in seawater at trace concentrations are chlorinated hydrocarbons, like chlorobenzene, dichlorobenzene, tri- and tetrachloroetheylen, as well as aromatic hydrocarbons including benzene, toluene and xylene isomers. Previous investigations have already demonstrated the feasibility of this set-up for measuring such compounds under real world conditions. This paper is focused on the evaluation of overlapping spectral features of different pollutants using chemometric algorithms, like principal component regression (PCR), for improved concentration determination in multicomponent seawater samples. The influence of the salinity and the turbidity on the spectra is also discussed. For the first time, pollutants in marine ecosystems can be determined on-line and *in-situ* with high precision, i.e. in the ppb concentration range. The development of the first mid-infrared sensing system for submarine applications significantly enhances the analytical methodology for marine monitoring and opens an entirely new field for spectroscopic chemical sensor systems.

### A new UV on-line photometer for low level $NO_x$ measurement from emission sources

*Bill Worthington[1] and Walter Fabinski[2], [1]ABB, Inc., Analytical Department, 843 N. Jefferson Street, Lewisburg, WV 24901-9509, USA, [2]ABB, Analytical, Stierstaedter Str, Frankfurt am Main D-60488, Germany*

Nitrogen oxides have a particular relevance as an environmental component with a global effect. The main sources are combustion engines and stationary combustion processes. The global reduction of $NO_x$ is mandated since progressing knowledge about the effect of nitrogen oxides on the climate and the biosphere are proven. In this effort a further reduction of the emission limits for stationary sources is sought which leads to measuring ranges of $\leq$10 ppm Knox. This new approach leads many users to look for alternative measuring techniques to meet the current challenge of these lower range requirements. In addition, a robust and reliable measuring technology with long maintenance intervals is always desired. This paper describes a new development of a new UV photometer for the measurement of $NO_x$ in applications in CEM and in the automobile industry. The technology is based on a UV-discharge lamp that delivers NO-specific UV-radiation. Since the emitted radiation is in resonance with the NO absorption lines, it results to high sensitivity and selectivity. This unique technique is called UV-RAS (ultraviolet resonance spectrometer). A four-channel signal processing gives a high stability. Since the measurement does not need any vacuum pump, ozonizer/de-ozonizer and auxiliary gases, it is robust and reliable and needs little maintenance. The analyser has the potential for a measuring range of 5–10 ppm $NO_x$. We will report about the results of tests run in the automotive industry and on turbine exhaust gases.

### HRMAS NMR as an analytical tool in combinatorial chemistry

*Guy Lippens, R. Warrass, P. Rousselot-Pailley, J. Martins, G. Chessari and J.-M. Wieruszeski, Institut Pasteur de Lille, Institut de Biologie de Lille, NMR Laboratory, Lille F-59000, France*

At the heart of the present day effort in drug discovery and development are the modern methods for stereoselective organic synthesis. A breakthrough has been the introduction of combinatorial chemistry, where large numbers of compounds are synthesized in a relatively short time. Less based on fundamental principles but rather on practical considerations, one could draw a division in this emerging field between solution and solid-phase methods [1]. One of the chemical advantages of solid-phase organic chemistry (SPOC) is the possibility of automation turning it into a method of choice for combinatorial chemistry. However, transposing the known solution reaction schemes to the solid-phase has not been straightforward in all cases. Therefore, it is now generally recognized that optimizing the reaction conditions is the most time consuming step. One of the disadvantages in this process is the lack of universal and rapid analytical methods for on-resin quantitative analysis of the solid-supported compounds, both at intermediate stages and of the final products, while avoiding the tedious and time-consuming procedure of cleave-and-analysis. For more complex reactions, the technique of High Resolution Magic Angle Spinning NMR (HR MAS) [2–4] is emerging as a new tool that finally could solve the analytical difficulties of SPOC. With exactly the same pulse sequences as in high resolution liquid NMR, time requirements and resulting resolution can equally be compared. We will discuss in this lecture our approach of HR MAS NMR, including (1) the diffusion filter we developed to allow work in protonated organic solvents [5], (2) different schemes to monitor reaction completion [6], (3) questions of impurity detection [7] and (4) the extension to macroscopic supports that make sample handling and hence automatic analysis more easy [8]. A number of examples involving less studied chemistries on the solid state will be shown to demonstrate the practical feasibility of the method.

1. Baldino, C. M., *Journal of Combined Chemistry*, 2 (2000), 89.
2. Fitch *et al.*, *Journal of Organic Chemistry*, 59 (1994), 7955.
3. Anderson *et al.*, *Tetrahedron Letters*, 36 (1995), 5311.
4. Pop *et al.*, *Tetrahedron*, 52 (1996), 12209.
5. Warrass *et al.*, *Journal of the American Chemical Society*, 121 (1999), 3787.
6. Warrass *et al.*, *Journal of Organic Chemistry* 2000, 65, 2946.
7. Rousselot-Pailley *et al.*, *Tetrahedron*, 56 (2000), 5163.
8. Rousselot-Pailley *et al.*, *Journal of Combined Chemistry*, 3 (2001), 559.

### Compliance and the automated pharmaceutical laboratory

*Kyle McDuffie, CSols, Inc., 7600 Southland Blvd, Orlando, FL 32809-6975, USA*

21 CFR Part 11 Electronic Records, Electronic signatures has provided an opportunity for dramatically increasing the level of information management automation used in Pharmaceutical R&D and Quality Assurance laboratories. Although LIMS have been in use for many years, most laboratories still maintain laborious paper transcription and review processes for analytical results. Results from instruments are printed on paper, pasted in notebooks and then entered into the LIMS. Once entered, they are printed again by supervision for review and then signed (and approved in the LIMS as well). Next generation instrument integration tools provide the level of automated workflow that are needed by laboratory and Quality Assurance staff to eliminate the need for paper calculation and transcription steps. These include typical calculations needed for content uniformity, dissolution and potency. In addition, they provide the level of robustness and regulatory compliance needed to defend the quality of the laboratory data. This presentation will focus on the key elements of 21 CFR Part 11 which apply to laboratories operating in GMP environments. Successful current and future approaches to addressing the compliance, quality and workflow automation challenges will be discussed.

### Speciation of arsenic, selenium and antimony using HPLC: atomic fluorescence spectrometry

*Derek W. Bryce, Warren T. Corns and Peter B. Stockwell, PS Analytical Ltd, Arthur House Crayfields Industrial Estate, Main Road, Orpington BR5 3HP, UK*

The speciation of arsenic, selenium and, to a lesser extent antimony, has gained in importance over the last decade. Differences in toxicity, bioavailability and integration of the different species in the biochemical cycle make the need for accurate speciation data imperative for further understanding. Arsenic is normally found as toxic inorganic forms in the environment, which may undergo biomethylation by microorganisms to relatively non-toxic organic forms such as DMA, MMA and arsenobetaine. Selenium's dual nature as both an essential and toxic trace element has been widely reported, but more recently it has been linked with cancer prevention when found as selenoamino acids. Antimony emissions from anthropogenic sources such as traffic and the production of storage batteries has lead to an increase in antimony levels in the environment where SbIII is less toxic than SbV, although organic antimony species may also be found. Speciation of these elements may be carried out using a variety of approaches, generally coupling an efficient separation technique with an element specific detector. Various HPLC approaches have been used, either using strong anion exchange or ion-pairing HPLC to separate the species of interest. Atomic fluorescence is ideal offering unrivalled sensitivity, linearity and freedom from interferences, making it ideal for speciation studies. Results will be reported for the speciation of the target analytes including information on separations and post column hydride generation. On-line oxidation and reduction of non-hydride forming species such as arsenobetaine and selenate will be discussed and applications for various samples will be shown.

### Zeeman atomic absorption spectroscopy with high-frequency modulation with a thin-walled metallic hollow cathode as a base of a new mobile monitoring system

*Victoria S. Vergizova, Alexander A. Ganeev, Stanislav A. Suprunovich, Alevtina A. Kovaleva, Ilya V. Shuvaev, St-Petersburg State University, Chemistry, Universitetskij, St Petergoff St-Pete 198904, Russia*

Nowadays, one of the most actual issues is the development of mobile system and tools allowing one to analyse heavy metals in a monitoring regimen. Such a tool should be of high analytical characteristics like traditional analytical techniques (high sensitivity, low detection limits) and provides mobility and besides monitoring analysis (low applied power, small size of technique, moreover absence or minimum of chemical samples pretreatment are desirable). One of the way to realize all of these features is to develop technique based on thin-walled metallic hollow cathode (TMHC) as atomizer with high-frequency modulation Zeeman atomic absorption spectroscopy (providing high sensitivity and analysis of complex samples without or with minimal sample pretreatment). In this technique, dry residue of solution is analysed. Owing to thermal-ionic mechanism and long trap time of analysed atoms in 'analytical signal forming zone' observed in TMHC low detection limits and high sensitivity compared with ones in graphite furnace take place. Moreover, due to self-clearing effect under discharge process TMHC has extremely low memory effect. Applied power is about two orders of magnitude lower than required to analyser with graphite furnace, discharge gas (Ar) consumption is also extremely low. There is possibility to analyse 2500 samples without TMHC changing in contrary to graphite furnace providing about order lower resource. All these advantages have allowed us to design new mobile technique allowing monitoring analysis of metals even in complex environmental and biological samples. Analytical parameters of new technique such as detection limits and matrix effects are presented. Detection limits for Cd, Mn and Cu are 0.3, 3 and 6 pg accordingly.

### Electronic nose as a quality-control tool in the pharmaceutical industry

*Jean-Christo Mifsud and Sophie Puech, ALPHA M.O.S, 20 Avenue Didier Daurat, Toulouse F-31400, France*

Flavours qualities and intensities attract an increasing level of attention from the pharmaceutical companies, particularly the one involved in the development of paediatric formulations. This industry invests heavily in instruments that can help it to ensure the quality and the consistency of their product. Different qualities and intensities of various flavours used in pharmaceutical formulations were successfully evaluated using a Fox4000 Electronic Nose (Fox4000 EN). The electronic nose has generated great interest in the analytical laboratories of the world's leading food, flavours, and fragrance companies as a fast, simple, and reliable method of aroma/VOC analysis. Over the past 5 years, the sensors arrays system have demonstrated their ability to produce information (instead of a stream of data) and the ability to transfer expert knowledge from the R&D and trained sensory panels into a production environment for quality assurance and control. Good discrimination and excellent reproducibility were obtained. The successful identification of acceptable quality and intensity of flavours in 'blind tests' have been obtained on unstressed and stressed samples for shelf life evaluation. Interbatch training sets of flavours were used to ensure that a representative batch variation was introduced into the model using custom identification method and also to ensure that the discrimination level of the acceptable, marginal and unacceptable flavours were sufficient when different batches were considered. Both Principal Component Analysis (PCA) and Discrimination Function Analysis (DFA) were used for data processing and identification. Good correlation between GC data, sensory panel and the Fox4000 EN were found. Finally, the principle of overall flavours intensity measurement was demonstrated using Partial Least Square (PLS) by showing data for flavours from various batches of reception and various intensities.

### A multichannel biochip with biofluidic module for real-time bioassays

*David L. Stokes, Guy D. Griffin, Leonard R. Allain, Dimitra N. Stratis and Tuan Vo-Dinh, Oak Ridge National Laboratory, Advanced Monitoring Development, 1 Bethel Valley Road, Oak Ridge, TN 37830-8050, USA*

This work describes the development of a biofluidics system that is coupled to a multichannel biochip sensor for real-time monitoring of bioassays. The biofluidics module features a single reaction chamber in which multiple bioassays can be performed simultaneously. In this technique, a sampling platform (e.g. membrane) is prepared off-line with a modified ink jet printing technology which allows rapid array printing of multiple, discreet capture probe zones or microdots on a single sampling platform. The sampling platform is then placed in the reaction chamber of the biofluidics module, and the pattern of capture probe microdots is aligned with an array of independently addressable biochip photosensors. The biofluidic system delivers samples and reagents for the ensuing bioassays. The system can accommodate both DNA and antibody-based assays, both of which have been performed on a single sampling platform. The reaction chamber also features a nichrome heating element and thermocouple probe for performing on-chip reactions at optimized temperatures. The biofluidics-based multichannel biochip detector has been applied to the detection of a variety of pathogens and analogues, including *E. coli* and *B. globigii*.

### Development of a semi-automated comprehensive extraction and multiple fractionation (S-ACEMF) METHOD—Part I: Evaluation of different solid phase sorbets and optimization of overall recovery

*Courtney D. Sandau, Andreas Sjodin, Mark D. Davis, Alyson L. Waterman, William Roman and Donald G. Patterson, Centers for Disease Control and Prevention, Toxicology Branch, 4770 Buford Hwy [1] Mai, Atlanta, GA 30341-3717, USA*

A semi-automated extraction method has been developed to increase our current sample throughput. The method employs solid phase extraction with automation on the RapidTrace® (Zymark Corporation). The current methodology has been evaluated for polychlorinated biphenyls (PCBs), hydroxylated PCB metabolites, methylsulphonyl PCB metabolites, persistent pesticides, polychlorinated naphthalenes and polybrominated diphenyl ethers. Sorbent evaluation: Nine different sorbents from several manufacturers were evaluated, which included C18®, ENV®, NEXUS® (all Varian, Inc.), OASIS® (Waters Corporation) and Chromabond® (Macherey-Nagel). Cartridges of all sorbents were packed in house to correspond to bed heights of 4, 12, 22 and 30 mm, respectively, in 3-ml cartridges. The weight of the sorbents used to obtain a certain bed height varied due to different densities of the sorbents. Duplicate samples were extracted for each bed height, using the RapidTrace®, employing the same extraction procedure. The procedure included (1) conditioning, (2) application of sample, (3) drying of cartridge and (4) elution of compounds of interest. All serum samples (2 ml) were pretreated using the same methodology employing formic acid (2 ml), sonication, and subsequent dilution with water (2 ml). Sorbent heights were plotted against absolute recoveries for each sorbent and analyte. Recoveries for highly lipophilic compounds such as decachlorobiphenyl (CB-209) were lower than recoveries for less chlorinated and less lipophilic compounds, such as 2,2,3,3′,4,4′-hexachlorobiphenyl (CB-153). However, recoveries increased for CB-209, to approximately 80% using a bed height of 30 mm for most sorbents, indicating how important these experiments are for determining the amount of sorbent needed for efficient extraction.

### Development of a semi-automated comprehensive extraction and multiple fractionation (S-ACEMF) method—Part II—Isolation and purification of PBDES, persistent pesticides, PCBs and PCB metabolites from serum

*Courtney D. Sandau, Andreas Sjodin, Mark D. Davis, Alyson L. Waterman, William Roman and Donald G. Patterson, Centers for Disease Control and Prevention, Toxicology Branch, 4770 Buford Hwy [1] Mai, Atlanta, GA 30341-3717, USA*

The comprehensive extraction method described by Sjodin *et al.* was coupled with a multiple fractionation method to allow the separation and quantitation of polybrominated diphenyl ethers (PBDEs), polychlorinated biphenyls (PCBs), persistent pesticides, halogenated phenolic compounds (including hydroxylated PCBs), and methylsulphonyl-PCBs (MSF-PCBs). Each of the fractions were isolated using specific interactions with different commercially available sorbents and were semi-automated using the Zymark RapidTrace® system (Zymark Corporation). The serum extract from the polymeric extraction sorbent was reduced in volume and separated into two fractions on activated (100%) silica gel—non-polar compounds (F1) and polar compounds which includes phenolic compounds, MSF-PCBs and polar pesticides (F2). Phenolic compounds in F2 were then derivatized to their methyl ethers and cleaned up further on silica/sulphuric acid columns to remove residual biogenics before mass spectral analysis. The non-polar fraction (F1) can be fractionated further on a carbon column to separate PCBs/persistent pesticides (F1a) from planar compounds, such as polychlorinated naphthalenes, dioxins and furans (F1b). Both fractions were purified when necessary before gas chromatography-mass spectral analysis. The final semi-automated method will be described along with the application of the method to human serum and plasma. The Zymark RapidTrace® allowed semi-automation of a complex method that significantly increased throughput of samples in our laboratory and also increased the number of analytes monitored due to the fractionation techniques described above. The potential of this method to replace the current vacuum manifold methods and to expand further to other classes of compounds, such as dioxins, furans and polychlorinated naphthalenes will also be discussed.

### Development of an integrated electrochemical immuno-analysis on microchip platforms

*Madhu Prakas Chatrathi, Joseph Wang, Alfredo Ibanez and Alberto Escarpa, New Mexico State University, Department of Chemistry and Biosciences, MSC 3C North Horse Shoe Drive, Las Cruces, NM 88003, USA*

Microfluidic devices have achieved rapid development in the recent years and continue to advance toward fully automated tool. The power and scope of micro-analytical systems can be greatly enhanced by performing highly selective biochemical reactions, in particular, on-chip immunoassays using highly specific antigen–antibody interactions. Previously reported microchip based immunoassays have been performed using either thermal lens microscopic (TLM) or laser induced fluorescence (LIF)

detection methods, however, there are no reports of analogous on-chip electrochemical immunoassays. One of the benefits of using electrochemical detection with microchips is the ability to maintain good detection limits with other benefits such as tuneable selectivity, high sensitivity and compatibility for further miniaturization. The present investigation focuses on a complete integration of individual steps of electrochemical immunoassay on chip. This includes precolumn reaction of alkaline-phosphatase labelled antibody with the antigen, electrophoretic separation of free antibody from antibody–antigen complex, followed by amperometric detection of 4-aminophenol product resulting from the reaction of 4-aminophenyl phosphate substrate with the enzyme tracer (alkaline phosphatase) in the post-column. In this paper, analytical protocols are described, conditions are optimized and data will be presented quantifying the performance. A remarkably low detection limit of $2.5 \times 10^{-16}$ g ml$^{-1}$ is obtained for the mouse IgG model analyte. This approach has a universal applicability as the target analyte of the assay can be changed simply by changing the antibody and is limited only by the availability of antibody for the analyte of interest.

### Chemometric analysis of two-dimensional gas chromatographic data

*Kevin J. Johnson, Bryan J. Prazen and Robert E. Synovec, University of Washington/Center for Process Analytical Chemistry, Department of Chemistry, Seattle, WA 98195, USA*

Two-dimensional gas chromatography (GC $\times$ GC) is applied to both a pattern recognition problem involving classification of jet fuel mixtures and to a trilinear partial least squares (tri-PLS) quantification of the composition of industrial naphtha samples. The use of two chromatographic columns with complementary retentive properties in GC $\times$ GC separations results in a significant increase in both peak capacity per unit time and selectivity over separations obtained with single-column GC methods. High-speed GC $\times$ GC analysis is accomplished through the use of short GC columns and high gas velocities as well as through the practice of following partial chromatographic peak resolution with multivariate chemometric analysis. This work is aimed at providing a GC system for rapid quantitative and qualitative analysis of complex samples such as process streams. In jet fuel classification studies, jet fuel samples are subjected to a high-speed 5-min GC $\times$ GC separation. An analysis of variance (ANOVA) based feature selection method is then used to identify and select chromatographic features relevant to a given classification between mixtures of different jet fuel types. In one experiment, 1% volumetric compositional changes in binary mixtures of JP-5 and JP-7 jet fuels are readily observed in scores plots from principal component analysis (PCA) of the selected features. In a second experiment, features adept at classification of jet fuel type but not sensitive to geographic origin of the sample are located in a data set consisting of GC $\times$ GC chromatograms of samples of three different jet fuel types. In the naphtha analysis study, trilinear partial least squares models are used to predict the aromatic and naphthene content of industrial naphtha samples. The tri-PLS models are constructed using chromatograms from six minute GC $\times$ GC separations of neat naphtha samples and corresponding reference values that were determined by a standard single-column GC method. The GC $\times$ GC/tri-PLS analysis method is shown to provide acceptable quantitative precision while using a separation time that is more than 15 times faster than the single-column standard method.

### Sample management for DNA testing laboratories

*Nick Townsend, Martin Waugh and Peter B. Mansfield, Autoscribe Ltd, 81 Technology Park Drive, East Falmouth, MA 02536, USA*

DNA technology has revolutionized applications such as paternity testing, forensics, clinical diagnosis and the detection of genetically modified organisms (GMOs). This success in turn has created a very large increase in the number of samples being processed by DNA testing laboratories and the associated logistical issues this can bring. In the last two decades LIMS have become established supporting the work of analytical and research laboratories but is it only recently that DNA testing laboratories been able to realize the benefits that LIMS can deliver. In this presentation we will describe a LIMS configured to meet the needs of DNA contract testing laboratories. We will describe how, via Web technology, clients submit samples for testing, specify the DNA testing profile and confirm order details. Once samples are received, bar code technology is used to track sample location and process steps including extraction, purification, amplification and identification. A very graphical and intuitive interface is provided for technicians allowing creation and editing of plate and electrophoresis gel plans using drag-and-drop techniques. The system supports the use of complex 'plating rules' providing a large degree of automation as to how primers, samples and reference standards should be pipetted onto the plates. A comprehensive audit trail is provided recording details of how samples are analysed including the run conditions associated with processing the plates and the transfer of samples from plates to gels. Once analysis is complete the LIMS automates the production of reports for the client and will interface with order processing systems to deliver an invoice for the work.

### Automated sample spotting on MALDI plates

*Alan Hamstra, Tim Hegeman and Luke Roenneburg, Gilson, Inc., 3000 W. Beltline Hwy, Middleton, WI 53562-1617, USA*

Sample preparation is a rate-limiting step in the analysis of samples with MALDI-TOF instrumentation. Plate preparation requires adding both sample and matrix, allowing them to form a homogenous mix and then drying. Knowing the limitations of hardware, sample concentration, sample volume and sample density helps predict sample thoughtput. Hardware is available with positional accuracy to $< 100\,\mu$m, however positional accuracy is only one parameter affecting the sample area occupied on a plate. MALDI plates come in a variety of sizes and configurations, placing requirements on sample application hardware. The volume of available sample imposes additional limits on hardware. The calculated theoretical diameter occupied by a 100 nl sphere is 726 μm. When placed on the plate surface, the drop compresses vertically and occupies a diameter > 726 μm. Plate surface chemistries, either hydrophobic or hydrophilic, also affect plate sample density. Matrix and sample surface tension and viscosity affect plate area occupied by sample. To pass through the small probe, the orifice matrix and sample must be free of any particulate or a probe will plug and dispense inaccurately. Required sample accuracy and carryover specifications place time limitations on plate preparation. Loading a 384-well plate with matrix requires only minutes, while rinsing probes between samples to prevent carry over requires from 20 to 60 min. A rinse cycle may take significantly longer than sample application and account for 50–90% of the time required to load a plate. Technology is available to dispense nanolitre volumes of sample, however there are limits on the sample volume, which can be aspirated. If the minimal aspirated sample volume is limited to microlitres, each dispense of nanolitres may waste microlitres. Hardware and software must recognize this limitation and take steps to conserve sample. I will present data on minimizing the area occupied by sample, optimizing sample volume and mass, and minimizing plate preparation time.

### Millisecond monitoring of fast transient signals by Hadamard transform time-of-flight mass spectrometry

*Joel R. Kimmel, Facundo M. Fernandez, Jose M. Vadillo and Richard N. Zare Chemistry Department Mudd Bldg, 333 Campus Drive, Stanford, CA 94305-4401, USA*

Hadamard transform time-of-flight mass spectrometry (HT-TOFMS) involves the modulation of a continuous ion beam using a pseudo-random sequence of pulses [1]. This sequence of pulses is applied using a charged particle modulator consisting of an interleaved comb of wires. Ions flying from the electrospray ion source to the flight chamber are 'gated' at this modulator following the Hadamard-type binary sequence. The applied sequence has an approximately equal number of zeros and ones, thus the duty cycle of the spectrometer is close to 50%. TOFMS has become the preferred detector for separation techniques used in bioanalytical chemistry such as CE and LC. As research continues, the obtained separation efficiency of these methods increases, making the eluting peaks sharper. To describe correctly the shape of these narrow peaks, detectors must acquire data at a rapid speed. The only means of increasing the spectral acquisition speed of a TOFMS without sacrificing mass range is to use encoding methods that eliminate the lag time between successive ion packets. In this presentation, we describe the ability of HT-TOFMS to scan transient signals in the millisecond time-scale. A brief theoretical background on how TOFMS can be multiplexed is given. Alternative Hadamard transform algorithms that improve the performance of slow multichannel scalers are discussed in terms of their effect on the spectral storage rate. The effect of acquisition systems' end-of-pass dead time is examined. The ability of HT-TOFMS to scan transient peaks of decreasing width is investigated for CE and pulsed electrospray ionization peaks. For femtomole injections of biomolecules, full spectra in a mass range of 1500 Da are obtained in 3.6 ms. Owing to the extremely large amounts of data collected, only state-of-the-art acquisition systems can be used for this task. Further increase in the spectral storage rate can only be achieved by further increasing the duty cycle of the instrument. The possibility of using two MCP detectors to produce a 100% duty cycle with no ion storage is discussed.

1. Zare *et al. Journal of the American Society of Mass Spectrometry*, **12** (2001), 1302.

### Quasi-continuous monitoring of process and waste streams by on-line inductively coupled plasma-optical emission spectroscopy

*Dirk Ardelt, Heinz Falk, Hendrik M. Smol and Hans-Joerg Waarlo, Spectro Analytical Instruments GmbH & Co. KG, Product Group ICP (T-3), Boschstrasse 10, Kleve D-47533, Germany*

Over the past decade, increasingly more sophisticated process analysers derived from 'mature' laboratory methods of instrumental analysis have found widespread use in the chemical process industry to cope with increasing demands for more efficient energy and material usage and better process control. Although many such applications still rely on rather simple sensors, an increasing amount of formerly laboratory based only methods, like infrared or Raman spectroscopy, X-ray fluorescence or magnetic resonance methods, are nowadays routinely applied as on-line systems. As diverse as these methods are, their fields of application are in the on-line process environment, which typically is divided among the distinction between the aggregate states of the medium to be analysed. Not surprisingly, one major field is the analysis of liquid process and waste streams. Considering laboratory use, inductively coupled optical emission spectroscopy (ICP-OES) probably is the most universal atomic spectroscopy method existing. Combining high flexibility, good sensitivity, wide dynamic range and fast measurement cycles, this method should ideally be suited for migrating into the process analytical field, especially regarding its relative 'maturity'. Despite of these findings, ICP-OES up to now has not found the expected use as on-line analyser, even where it outperforms other methods under laboratory conditions. Using recently installed systems for wastewater and process stream monitoring as examples, we will discuss probable reasons for the rather slow past development of on-line ICP-OES and examine how far these reasons have been or can be overcome. Relevant analytical and technical figures of merit for on-line applications shall be deduced. Furthermore, modern multichannel detection ICP-OES allows for using sophisticated algorithms (e.g. chemometrics) for data evaluation, in this case resulting in higher degrees of automation and system availability. Accounting for such advances, we will try defining a 'state of the art' for ICP-

OES as process analytical method and highlight future development areas.

### A new stand-alone autosampler for high-throughput HPLC and HPLC/MS

*Curtis R. Campbell, Susan M. Steinike and Kara M. Merkle, Shimadzu Scientific Instruments, LCMS Applications, 7102 Riverwood Drive, Columbia, MD 21046-1245, USA*

Fast LC and fast LCMS methods have been developed to meet the demands of combinatorial chemistry and drug discovery. Now more than ever is there a corresponding requirement for ultra-fast sample introduction in LC and LCMS instruments along with the most stringent requirements for zero carryover and optimum sample repeatability. In this communication we describe the performance of a newly designed stand-alone autosampler for HPLC and HPLC/MS applications featuring a 15-s injection cycle for 10-μl injections, excellent sample repeatability and the lowest carryover currently attainable. The unit can function independently, allowing interface with a variety of instrument models in a variety of venues including combinatorial chemistry, drug discovery and high-throughput screening.

### Ultra-fast liquid chromatography: the importance of system temperature

*Jon D. Thompson and Peter W. Carr, University of Minnesota, Department of Chemistry, 207 Pleasant Street SE, Minneapolis, MN 55455-0431, USA*

It is very important that the speed of HPLC be increased. Improving throughput for stability analysis, quality control assays and dissolution testing are all key motivations for improvement in separation speed in the pharmaceutical industry. Unlike CE, HPLC does not lend itself well to multiparallel analysis because the cost is prohibitive. Recent work in this laboratory has focused on the theory of high temperature on analysis time in LC. Disregarding selectivity considerations, we have shown that the 5–10-fold decrease in eluent viscosity that comes from working at very high temperature (180–200 °C), and the concomitant increase in analyte diffusitivity, combine dramatically to decrease the time needed to generate a theoretical plate. The lower viscosity at elevated temperature decreases the pressure drop across the column and allows higher linear velocities to be reached as the pump's pressure limit is approached. Simultaneously, faster analyte diffusion at higher temperature improves efficiency at high linear velocity conditions compared with the efficiency at lower temperatures at the same velocity. In this presentation, we will show how the theory of speed guides column selection and system design. We will show that at high temperatures, 10 gradient runs can be done in 40 min on columns of conventional geometry. We will also show that dramatic improvements in stability—indicating assay throughput—can be achieved at elevated temperature using conventional equipment.

### Development of a high-throughput analysis infrastructure for combinatorial materials science applications

*Radislav A. Potyrailo, General Electric, Corporate Research and Development, PO Box 8, Schenectady, NY 12301-0008, USA*

High-throughput methods for materials science combine a parallel or combinatorial materials synthesis and processing with an automated screening and data management tools. They can rapidly optimize molecular properties and process conditions that are difficult to predict using existing knowledge. From an analytical chemistry perspective, this leads to major challenges in developing rapid analysis techniques that can deal with large numbers of small samples required to construct combinatorial libraries. To meet these analytical challenges in the discovery of new materials at General Electric, a high-throughput analysis infrastructure was established at GE's Corporate Research Center. This presentation will describe the strategy taken in the development of this infrastructure. For high-throughput analysis of materials properties from a wide variety of projects, analytical instrumentation consists of interchangeable modular subsystems coupled with the new data-processing capabilities. Examples from several projects will illustrate the broad applicability of the developed high-throughput analysis instrumentation.

### High-throughput analytics employing automated sample preparation and the integrated use of HPLC-DAD, HPLC-DAD-MS, GC-MS and flow injection NMR

*Winfried Etzel[1], Stefan Beeck[1] and Wolfgang Gau[2], [1]Bayer AG, Agrochemical Division, Chemical Research GEB 6530, Leverkusen D-51368, Germany, [2]Bayer AG, Landwirtschaftszentrum Monheim, Alfred-Nobel-Str 50, 6530, Monheim D-40789, Germany*

The establishment of automated and parallelized synthesis and micro-scale reactions in the chemical synthesis laboratories of the Agrochemicals Division at Bayer created an increasing demand for analytical information (purity, structure, physicochemical properties). With the availability of flow injection NMR systems (BEST-NMR) the use of the same sample format on different analytical instruments was possible. This fact encouraged us to realize an integration of different analytical techniques (NMR, HPLC-DAD, HPLC-DAD-MS, GC-MS) in one service unit. The optimization of the flow NMR system for the use of different organic solvents will be demonstrated and the quality of the spectra and the throughput of samples discussed. In addition, the results and advantages of an integration of different analytical techniques will be presented. Working with barcodes and microtitreplate (MTP) technology were introduced. Electronic laboratory journal, sample preparation robotic system and analytical instruments were connected together via a laboratory information and management system (LIMS). This resulted in a simplified workflow in the chemical research laboratories and in the analytical department. The throughput of samples and measurements was doubled without raising the number of technicians.

### An automated system for combined sample preparation and analysis for high-throughput-screening

*Kerstin Thurow, Agnes Schubert and Christian Wendler, University Rostock, Institut Automatisierungstechnik, R-Wagner-Strasse 31, Rostock-Wamemunde D-18119, Germany*

The use of combinatorial methods in chemistry and life science has been developing rapidly within the last years. One of the 'bottlenecks' in synthesis control in pharmacy and life sciences is still the characterization and speciation of biotechnological reaction products using chromatographic methods in combination with specific detection systems. Often before analysis, a complex sample preparation is required which might include cleaning steps, dilution or even derivatization procedures. Existing systems are not flexible automated solutions and do not have flexible material and information interfaces. Thus, the objective of our investigation was the development of an automated system for combined sample preparation and analysis being used for organic synthesis. The system used is a commercial liquid-handling station (CTC CombiPal) which has been equipped with different trays for heating, filtration, mixing etc. and has been adapted to different analytical devices such as gas or liquid chromatographic systems or mass spectrometers. Technically, the system is controlled by a digital computer operating both chromatograph and sampler as different tasks in the WINDOWS-NT 4.0 operating system. The sample preparation task allows the application of user specific methods. Sampler and chromatograph communicate for transmitting remote START/STOP-information via hardware handshake lines. Because of the flexible system strategy, the system can be set up easily for different applications. The system developed allows the fully automatic analysis of samples to run in parallel with the sample preparation. It can be used as a stand-alone system or can be integrated in complex robotic systems. Motivation for such a combination is to reduce testing costs further by increasing throughput and reducing manual intervention. Other advantages include, for example, reducing the delay between the analysis and the sample preparation. The presentation will outline the technical details of the system developed and will show the use of the system for high-throughput screening applications in the field of combinatorial catalysis, chiral determinations and synthesis control.

### Modification of OSHA GC methods for continuous area monitoring

*John N. Driscoll, Process Analysers, LLC, 25 Walpole Park South Drive, Walpole, MA 02081, USA*

OSHA has developed hundreds of GC methods for analysing organic compounds in the workplace as part of the standards process. These methods were developed to provide analytical methods for samples collected on adsorbent tubes in the workplace for a wide variety of industries. Typical sample volumes collected range from 250 ml (at the ceiling value) to 12 litres (below the TLV) of sample collected on the adsorbent tubes. Once the sample (250 ml) is diluted, it is equivalent to injecting a 1 $cm^3$ sample at approximately 5× the TLV. For area monitoring, we would like to have a detection limit that is 1/20th of the TLV or PEL. If these detectors were used in an Automatic GC for Area monitoring, many would have difficulty detecting half the TLV for those compounds with low TLVs. In other words, many of these methods would be 'sensitivity challenged' because of the use of the flame ionization detector (FID). These OSHA GC methods can still be used but it is clear that a more sensitive method (or a concentrator for the FID) is needed to obtain the required sensitivity. The photo-ionization detector (PID) is > 50 times more sensitive than an FID for aromatic compounds. This will allow a smaller sample (0.1–3 $cm^3$) to be used and still have adequate sensitivity for the method. In addition, the selectivity of PID can be varied by choosing a 9.5, 10.2 or 11.7 eV lamp. The sensitivity of the FID can be increased via an on-board automatic concentrator that can improve the detector sensitivity by 10–100-fold. The compounds to be concentrated can be optimized by switching the trap materials. Another difficulty that occurs at low ppm or ppb levels with polar species is adsorption or reactivity on/with surfaces of the sampling system and lines. This results in serious problems with reproducibility and accuracy. Examples include amines, phenols, organic and inorganic acids, pesticides, etc. A permeation tube or other diffusion device can be used to condition the entire system and prevent adsorption of polar or reactive compounds. We will evaluate a number of methods for amines, sulphur compounds, chlorinated hydrocarbons and diphenyl oxide. We will compare these results of the FID/NPD detectors with the photo-ionization detector (PID) mounted on an automatic GC for area monitoring. This should provide an alternate method that has more sensitivity and/or selectivity and does not require support gases.

### Fast gas chromatography with conventional instruments using direct resistively heated capillary columns

*Paolo Magni, Giacinto Zilioli and Riccardo Facchetti, ThermoQuest Italia SpA, Research and Development, Strada Rivoltana, Rodano, Milan I-20090, Italy*

In gas chromatography, there is an increasing demand for significant reductions in analysis time. However performing high-speed separations while keeping enough separation efficiency is a difficult task. The combined use of fast temperature programming and short narrow-bore columns may provide an optimal solution, particularly for samples containing simple mixtures with a wide boiling point range. For some applications a sufficient separation can be achieved even in < 1 min provided that, in the same time, the column is raised to an appropriate final temperature. The system presented in this paper, consisting in a directly heated capillary column module mounted in a conventional GC instrument, permits one to achieve very fast temperature programming rates (as high as 20 °C/s), which cannot be obtained with the use of conventional air circulating GC ovens. The tremendous heating rate power of the device permits one to reduce the tight bandwidth sample injection requirements otherwise dictated by the small internal diameter of the column. The module, containing the capillary column, the heating element and the temperature sensor, is housed in the GC oven and connected to standard GC split-splitless injector and detection systems, just as any conventional capillary column. Results obtained with both split and splitless injection techniques are examined using Flame Ionization Detector and Time of Flight Mass Spectrometer. The attached chromatogram shows an example of fames analysis, ranging from n-C7 to n-C22 frames, performed in < 1 min. The separation was obtained using a 5 m, 0.1 mm i.d., 0.1 μm film thickness RTX-5 column with a temperature-programming rate of 5 °C/s (from 70 to 300 °C). Others applications of the fast GC accessory in the chemical, petrochemical, environmental, and food and flavours fields are presented.

### Screening for environmental contamination using liquid chromatography with mass spectrometry detection

*Jim Krol and Kate Yu, Waters Corporation, 34 Maple Street, Milford, MA 01757-3696, USA*

When assessing unknown contamination of an environmental sample, where does the chemist begin? Which validated method is appropriate, a specific analyte method or several different analyte methods? What if the validated method reports no contamination; productivity decreases, analysis cost increase and the contamination analyte remains unknown. Liquid chromatography (LC) has the capability to retain and separate numerous analytes based upon their chemical properties, but conventional PDA-UV detection has limitations in seeing all the analytes. For analyte identification, retention time alone is not sufficient. Chromatographic analyte coelutions will exist in complex samples and compromise UV spectra making identification and quantitation marginal. If the analyte is UV inactive, analyte identification and quantitation are impossible. Mass spectrometry (MS) is a more universal, yet specific detection technique that detects a significantly greater number of environmental analytes, such as carbamate and urea based pesticides and herbicides using positive electrospray. Combined with gradient reverse phase chromatography, an environmental sample can be screened for numerous analytes. The simultaneous use of retention time, $m/z$ mass spectra and PDA-UV are used as qualitative variables for analyte identification. However, the low ppb semiquantitative results need to be confirmed by specific validated methods. This presentation will present a novel carbamate and urea analyte screening method approach. The critical link is the reliability of the MS processing method, chromatographic reproducibility, and the percentage of false-positives/false-negatives. Wastewater and ground water will be the evaluated matrices.

### Miniaturized flow-through surface plasmon resonance detector for the study of protein–protein interaction dynamics

*Rebecca J. Whelan[1], Thorsten Wohland[1], Richard N. Zare[1], Lars Neumann[2], Jacqueline Steenhuis[2] and Brian Kobilka[2], [1]Department of Chemistry, Mudd Chemistry Building, Stanford, CA 94043, USA, [2]Departments of Medicine and Cardiology, Beckman Center, Stanford, CA 94305, USA*

Surface plasmon resonance (SPR), which senses changes in the refractive index of a dielectric medium adjacent to a thin gold film, is a powerful tool for label-free studies of biological molecules and their interactions in real time. We employ SPR to study the interaction of the G-protein-coupled beta-2 adrenergic receptor with other molecules, including receptor agonists and antagonists, kinases, and G. protein. The most relevant information comes from receptors that are immobilized on the surface in a uniform way. To this end, we have developed a novel mutation of the receptor, with an additional cysteine at the extracellular terminus. Biotinylation of this mutant receptor and use of conventional biotin/avidin binding fixes the receptor to the gold film of the SPR sensor with excellent uniformity. An alternate immobilization protocol uses an antibody (M1) directed against the receptor's amino terminal FLAG tag. The receptors retain their function after immobilization, as confirmed by fluorescence microscopy studies. After introduction of a reaction partner through a flow cell (operating with or without continual flow), information can be obtained about the extent of interaction, binding kinetics, and pharmacology. Other biological interactions we have investigated include antigen/antibody, glycoprotein/lectin, and ssDNA/ssDNA. Regardless of the analyte system, maximum sensitivity and efficiency are achieved by minimizing the volume of sample above the sensing surface. To minimize the required sample, we have developed a miniaturized flow cell. This small flow cell also opens the possibility of coupling SPR with a miniaturized separation platform such as CE or micro-HPLC.

### Determination of sulfhydryl residues in cysteine-rich metalloproteins using flow-injection quartz crystal microbalance

*Alejandro Lopez Briseno, Alfred J. Baca, Fayi Song and Feimeng Zhou, California State University, Chemistry and Biochemistry, 5151 State University Drive, Los Angeles, CA 90032-4226, USA*

Metallothioneins (MTs) are low-molecular weight proteins having amino acid compositions rich in sulphur which strongly bind to metal ions such as Zn, Cd, Cu and Hg. Extensive research has been conducted concerning the two types of redox groups (metals and mercaptide groups) in these molecules. Particular emphasis has been aimed at determining the number of sulfhydryl groups involved in the electron transfer reactions of metallothioneins. However, less attention has been directed in the determination of sulfhydryl groups that are not directly involved in the redox properties of these proteins. We report here our recent efforts in determining the number of sulfhydryl groups that directly participate in the redox reactions of Metallothioneins. It was found that approximately four cysteines per MT molecule are involved in the cysteine-mercury thiolate formation. This was accomplished by combining two well-known techniques: the electrochemical quartz crystal microbalance (EQCM) and the inductively coupled plasma atomic emission spectrometry (ICP-AES). Another objective of this study will be to determine the availability of non-participating cysteine groups by signal amplification through chemical reaction to convert sulfhydryl groups into easily detectable moieties. A conceivable method of accomplishing this is by selective modification onto cysteine moieties acting as probes to methoxy-polyethyleneglycol maleimide (MPEG-MAL). The quartz crystal microbalance (QCM) will be used for gravimetric analysis of the sulfhydryl groups.

### Profiling compounds for their solubility properties

*Christopher A. Lipinski, Pfizer Global Research and Development, MS 8118-220, Eastern Point Road, MS 8118-2, Groton, CT 06340, USA*

Oral absorption depends on adequate solubility and intestinal permeability. Poor aqueous solubility due to high lipophilicity is fairly easy to predict computationally and to avoid. However, poor solubility due to crystal packing forces is actually more common. High-throughput solubility assays can be designed in two different ways. In one method a DMSO stock solution of the compound is serially diluted with aqueous medium and the precipitation point is determined by a turbidimetric assay. An advantage is high assay capacity and the ability to construct a relative solubility ranking of compounds. A disadvantage is the high percentage of DMSO in the aqueous. High DMSO content also occurs if 5 or 10 $m_M$ DMSO stock solutions are added to an aqueous medium. An alternative method that we use preserves the connection to a thermodynamic solubility assay to a greater extent. The design is based on identifying the critical thermodynamic solubility range for the most commonly encountered type of compounds. Our assay mimics the early discovery method by which a biologist orally doses an animal. Compound is added to the aqueous as a 60 $m_M$ DMSO stock solution thus keeping the final percentage of DMSO quite low. Turbidimetric solubility values always numerically exceed thermodynamic values, are very relevant to early discovery, but should never be used in the late discovery setting. Our two complimentary turbidimetric solubility assays have a throughput of 10 000 compounds per year. A flow cell assay measures solubility in the range 5–65 μg/ml, the critical range for the typical poorly soluble heterocycle with average permeability and average projected clinical potency. A plate reader assay measures the higher solubility range 50–500 μg/ml, the critical solubility range for the typical poorly permeable peptido-mimetic. Poor aqueous solubility has considerable relevance to experimental permeability studies and can be a considerable cause of experimental error even at a permeability screening dose as low as 10 $\mu_M$.

### Process monitoring of the moisture and finish-on-fibre of textile products using a portable near infrared analyser

*James E. Rodgers*[1], *Rafael L. Barraza*[1], *Panitan Sukpaladisai*[2] *and Charles M. Horton*[3], [1]*Solutia, Inc., 3000 Old Chemstrand Road, Cantonment, FL 32533-8926, USA,* [2]*Solutia, Inc., Highway* 20 West, Decatur, AL 35609, USA, [3]*Solutia, Inc., 1515 Highway 246 S., Greenwood, SC 29646-8402, USA*

The moisture content and the quantity of finish applied to the surface of textile fibres (Finish-On-Fibre, or FOF) are often critical process and quality control variables, for they can significantly impact physical properties, manufacturing processes, quality, and productivity. A recurring problem for textile bobbin products is the rapid detection and identification of outlier moisture and FOF bobbins during production. Non-contact, at-line moisture and FOF measurements directly on the bobbin in manufacturing would result in improved yields, process monitoring, and Quality Assurance. In this work we establish the feasibility of using a portable Near InfraRed (NIR) moisture analyser (Kett KJT100) to measure moisture directly on nylon tire and carpet yarn bobbins in manufacturing and demonstrate the feasibility of a subjugate process monitoring measurement of bobbin FOF during spinning. NIR moisture calibrations were developed for numerous tire and carpet products (different yarn type, size, finish, etc.) at three locations (laboratory, lag area, spinning). The NIR method successfully monitored moisture differences between bobbins in the laboratory and manufacturing areas. In spinning, a strong correlation was observed between NIR bobbin moisture and FOF. Subjugate FOF calibrations for tire and carpet products were developed in spinning, and excellent agreement was observed between the NIR and reference FOF results. Of critical importance to manufacturing was the rapid at-line identification of several ‘outlier’ bobbins, preventing their contamination of downstream processes. The impacts of yarn parameters, measurement location, and environmental and operational conditions on the NIR results were slight.

### Novel applications of Raman spectroscopy to process monitoring and materials’ characterization

*Brian J. Marquardt, CPAC, Center for Process Analytical Ch, FJ-20, Seattle, WA 98195, USA*

This presentation will focus on the use of Raman spectroscopy for the analysis of solid samples (powders, slurries, etc.) with emphasis on on-line process analysis applications. A novel high-precision Raman probe for on-line process analysis will be described. The unique design of the Raman probe provides enhancements in measurement precision by increasing the reproducibility and accuracy of optical sampling of high solid content samples. The probe has been proven an effective sampling interface for the analysis of powders, suspensions, slurries, particles and solids. The ease of use of the Raman probe and the increased sampling precision has lead to its use in various proof of concept and on-line process analytical applications. These applications include dry powder–powder mixing efficiency, coating thickness measurements, solvent drying analysis, reaction monitoring and various other analytical processes. In this presentation I will discuss the physical and optical design of the Raman probe and demonstrate its applicability as an on-line sampling tool.

### Quantitative in-line measurements of paper coatings by near infrared

*Jon G. Goode[1], Qian Wang[1], Angela Schmidt[2] and Helmut Weiler[2], [1]Bruker Optics, Applications, 19 Fortune Drive, Billerica, MA 01821-3923, USA, [2]Bruker Optik, Near Infrared Applications, Rudolf-Plank-Strasse 23, Ettlingen D-76725, Germany*

Leading manufacturers in the paper industry are looking for in-line methods to determine the physical properties, perform component analysis and determine applied coating weights in paper. The MATRIX-E, an in-line process control FT-NIR spectrometer, makes possible a non-contact diffuse reflectance measurement with a large sampling area. We will discuss an on-line paper application to determine the silicone coat weights on labelstocks. This is currently performed in the laboratory by a time-consuming X-ray fluorescence method. The aim was to achieve an absolute value for the deviation from the target of 1 g/m$^2$ during continuous paper production at velocities of approximately 400 m/min. Concentrations of silicone between 0 and 2 g/m$^2$ on various paper substrates were included in a quantitative model and influences from the uncoated paper type due to supplier, colour, opacity, area densities, precoatings as well as different compounds of the silicone agent were investigated. It was found that all these factors could be included in a single PLS model. The fact that elemental silicone is present in clay-coated papers was found to be of no consequence to the measurements with MATRIX-E. Moreover, during in-line installations it was found that the variation of the moisture content in the moving paper due to variable machine velocities as well as the reflecting material of the cylinder had to be considered. It will be shown that the result of the in-line calibration has the same prediction capability as laboratory scale results (root mean square error of cross-validation, RMSECV = 0.034 g/m$^2$).

### Identification of protein folding subunits by ion mobility-mass spectrometry

*Brandon T. Ruotolo, Kent J. Gillig, Earle G. Stone and David H. Russell, Texas A&M University, Department of Chemistry, PO Box 30012, College Station, TX 77842-3012, USA*

There have been several theories proposed as to the mechanism of folding processes in proteins. Among them, the idea that certain peptides segments of a protein exhibit intrinsic stability and contain the site(s) of helix nucleation within the protein, i.e. autonomous folding subunits, has been proposed and observed in a few isolated cases. However, with the introduction of relatively new methods for probing the conformation of biological molecules in the gas phase, such as ion mobility spectrometry, additional information can be obtained on the validity of this hypothesis. Proof-of-concept experiments have been performed on tryptically digested proteins, which are then screened by MALDI-IM-TOF MS. For example, a peptide signal from a tryptic digest of horse heart myoglobin was observed to deviate by more than 10% in total drift time from the other peptides in the IM-MS map. The peptide was sequenced using tandem mass spectrometry and identified as the majority of the E helix of solution phase myoglobin. This peptide was found to exhibit helical structure in simulated annealing molecular dynamics simulations. This presentation will focus on our recent efforts to probe the ability of MALDI-IM-TOF MS to screen for the presence of these extraordinary peptides. The updated screening protocol involves digestion with a number of different proteolytic agents and in different solvent systems in order to produce a more complete map of the protein under investigation. These results will be discussed in light of insight gained on protein folding mechanisms.

### Automatic HPLC method development with intelligent peak tracking

*Wolf-Dieter Beinert[1], Volker Eckert[1], Reinhold Spatz[1], Sergey Galushko[2] and Irina Shishkina[3], [1]Merck KGaA, Scientific and Laboratory Products, Frankfurter Str 250, Darmstadt D-64271, Germany, [2]Institute of Bioorganic Chemistry, National Academy of Sciences, Fine Organic Synthese, Im Wiesengrund 49-b, Muehltal 64367, Ukraine, [3]Vsevolod Tanchuck, Institute of Bioorganic Chemistry of National Academy of Sciences, Murmanskaya 1, Kiev-94, Ukraine*

An automated expert system has been developed to search for optimum conditions in HPLC. Controlled by an artificial intelligence module, the system provides fully automated unattended method development in reversed-phase HPLC for mixtures of compounds and can search for the optimum concentration of an organic modifier for isocratic separation, for the best linear and multisegment gradient profiles and for the temperature optimum. For proper peak tracking, the first version of ChromSword Auto required single standards of the compounds to be separated. However, often one faces the situation that not for all compounds to be separated suitable standards are available. An example is the analysis of impurities in a product, where usually only a few (if any) of the impurities are available as pure substances. This is usually the case with pharmaceutical quality control samples for purity and stability tests. Therefore, ChromSword Auto has been equipped with intelligent peak tracking capabilities. Applying advanced mathematical procedures for peak assignment, it is now possible to perform the optimization procedure with the sample mixture. The number of standards necessary for the optimization procedure can be reduced to just two, regardless of how many compounds are present in the mixture. The objects of the automated optimization procedure are: (1) separation of the mixture into a maximum number of peaks (e.g. search for impurities) or separation of target compounds; (2) optimum resolution; and (3) minimum analysis time In this way it is possible automatically to optimize HPLC separations of chemical and pharmaceutical samples where only a few substances (e.g. the active compounds) are available as standards. ChromSword Auto thus makes possible dramatic timesavings.

### The virtual assistant—a new HPLC system with expert knowledge and best performance

*Reinhold E. Spatz[1], Wolf-Dieter Beinert[1], Masahito Ito[2], Honori Kaji[2] and Richard Jack[3], [1]Merck KGaA, SLP Chromatography, Frankfurter Str 250, Darmstadt D-64271, Germany, [2]Hitachi Instruments Ltd, Biosystems, Hitachi-Naka 312-8504, Japan, [3]Hitachi Instruments, Inc., 3100 N. 1st Street, San Jose, CA 95134-1942, USA*

HPLC is by far the most used instrumental technique in the analytical laboratory. The instrument specifications continuously need to be adapted to new analytical requirements such as different column dimensions, high sample capacity, fast sample throughput, latest governmental regulations or state-of-the-art information management. The paper describes the concept of the newly developed modular LaChrom Elite HPLC instrument system. It is suitable as well for standard HPLC applications as well for special application segments in HPLC, like semi-micro HPLC or high-throughput separations with monolithic stationary phases. The same system can be modified by the user without compromising specifications for the application field in target. This flexibility is due to a new hardware concept and due to integrated unique software options that act like a 'virtual assistant' in the back-ground to deliver highest expertise whenever necessary for a certain application. Typical examples that require optimized hardware are semi-micro HPLC with 1 mm i.d. columns or High Throughput HPLC with monolithic columns and flow gradients in the 10 ml/min flow rate range. Typical examples for Virtual Assistance are integrated software modules such as 'ChromSword Auto' for fully unattended HPLC method development or 'AutoValidation' for automated Operation and Performance Qualification (OQ, PQ) of the HPLC system for regulated Quality Control Laboratories. The paper will describe the new hardware and software concept. Many evaluation results and typical applications will prove the high technical level of this new development.

### An automated software approach to analytical method validation

*Michael E. Swartz and Patricia A. Fowler, Waters Corporation, Pharmaceutical Marketing Lab, 34 Maple Street, Milford, MA 01757-3696, USA*

Method validation is a tedious process performed to determine if an analytical method meets the requirements for its intended purpose. In the regulated laboratory, method validation may take many days to perform the necessary analytical tests. Data reduction and the statistical analysis of results performed can be a very time consuming process. There is also a greater possibility of introducing error when calculations are performed manually. With the use of automated software to perform these calculations, method validation is much faster and easier, with less chance for error. In this presentation, we will show how an analytical method is validated using automated software. Chromatographic results are directly accessed from a relational database bypassing manual intervention. Statistical calculations are performed automatically and a report generated showing the results of the analyses from the Student, Cochran, Dixon, and Fisher Tests. Graphs are generated representing the results of the statistical analysis. In addition, we will show that the data reduction and statistical calculations necessary to validate the method, complete with the necessary documentation and report generation, are completed in significantly less time.

### A collaborative environment for developers of scientific software

*Deborah A. Kernan, Victoria Rafalovsky and Ty Abshear, Bio-Rad Laboratories, Informatics Division, 3316 Spring Garden Street, Philadelphia, PA 19104-2552, USA*

Bio-Rad's KnowItAll™ Analytical System offers an integrated environment for analytical techniques, such as IR, H-1 and C-13 NMR, UV/Vis, GC, MS, Raman, and NIR. Within this system, chemists can perform multiple tasks within the same interface, using software 'plug-ins' that reside within the main KnowItAll architecture. This design allows the user to easily transfer information from plug-in to plug-in without having to open another program. Because of this unique design, new third-party software plug-ins can be quickly and easily incorporated into the KnowItAll architecture. The philosophy of this unique architecture is one that built upon the ideal of getting solutions to scientists faster and giving them more options. Thus, through collaborations with database content providers and third-party software developers throughout the world, the system grows and adapts to meet the changing needs of the scientific community. For those parties with existing software that may fit within the content of the system, Bio-Rad has developed a simple Software Developer's Kit to convert software into a format that will 'plug into' and work within the KnowItAll system.

### Development and validation of analytical software in a regulated environment

*Kevin Bynum, Gillian Raymond, Lane Gehrlein and Philip Palermo, Purdue Pharma LP, Pharmaceutical Analysis, 444 Saw Mill River Road, Ardsley, NY 10502-2605, USA*

The development and validation of an analytical software package for use in the collection of cGMP compliant data will be discussed. The system has been designed to collect data to support drug development, stability testing, and release testing in the Pharmaceutical Industry. The software is designed to collect dissolution data from a UV probe fibre optic dissolution system. The software, written in JAVA and C++, uses an Oracle database to ensure data integrity and security. The design features, which make this software 'validatable' will be discussed. The validation testing and implementation of the system on our corporate network will be discussed in detail.

### Validation Manager 2.0: new developments in computer-assisted assay methods validation

*Jean-Marc Roussel[1], Michel Righezza[2] and Wolf-Dieter Beinert[3], [1]Merck Eurolab S.A.S., 201 Rue Carnot, Fontenay Sous Bois F-94120, France, [2]Antenne Scientifique Universitaire de Chartres, LBC, 21 Rue De Loigny La Bataille, Chartres F-28000, France, [3]Merck KGaA, SLP Chromatography, Frankfurter Str 250, Darmstadt D-64271, Germany*

For laboratories willing to meet the requirements of ISO 17025 regulations, analytical assay methods validation is an step important to consider. Recommendations for these method validations have been announced during the fourth International Conference on Harmonization (ICH 4) and the corresponding statistical calculation procedures are described in the ISO guidelines (i.e. ISO 5725, ISO 8466). Based on these recommendations, we have developed an assay methods validation software which allows validation planning, calculations and reporting according to the authorities requirements (USP, EP, FDA). Validation step consists in the study of the method characteristics as described in the guidelines and uses well-known and efficient statistical tests such as Dixon, Fisher, Student or Cochran. Although these statistical tools are frequently used in the analytical laboratories, most questions come when preparing the validation planning or determining the correct calculation configuration to be used. In order to help the analyst, we have implemented in the software ready to use validation templates which include, for different analytical techniques, predefined statistical tests confidence levels and default number of analytical results per validation characteristics, all these in conformity with the international recommendations and regulations. A validation document preparation wizard may be used in combination with these templates, in order to prepare the detailed validation planning in the easiest way. Electronic data security has become of the utmost importance, in this respect Validation Manager software proposes to the user a FDA compliant configuration in which every single addition or modification in data or result is fully documented and saved in a method logbook. Each versions of the validation document is saved in a specific folder and cannot be overwritten. Each data modification and calculation creates a new results version, ensuring full data integrity. Moreover, in order to avoid transcription mistakes, an automatic data recovery function has been developed allowing direct copy of information from the Hitachi D-7000 HSM and SSI EZ-Chrom Elite chromatography data systems. Using analytical data from our laboratory, the presentation will describe the different steps of method validation using Validation Manager. The next future of this development project, including the use of experimental designs for robustness study will also be presented.

### Advanced automation interfaces: new toolkits for adding instrument control to a chromatography data system

*Dario Fiore and Kristi McKiney, Scientific Software, Inc., 6612 Owens Drive, Pleasanton, CA 94588-3334, USA*

One of the greatest limitations of chromatography data systems is in the area of true (interactive) instrument control for different types of instruments from disparate manufacturers. Because instrument manufacturers are in business to sell instruments, many feel it is not in their best interest to provide control for competitive instruments. Yet this means the user must learn to operate multiple data systems in a single lab, many of which might not be the data system of choice. Hundreds of man-hours are wasted in learning, maintaining, and validating different data systems that essentially do the same thing—data acquisition, analysis and instrument control. Many companies (both data system users and instrument manufacturers) are realizing the time and cost benefits of using open-architecture data systems where instruments of many brands are supported. Although adding control for an analytical instrument is not a task for the everyday user, new tools are now available that facilitate the interfacing of instruments to the EZChrom Elite Chromatography Data System, making it much easier and faster for instrument manufacturers and large companies to create an instrument control interface. The Toolkit automation interface provides the tools required for a qualified company to add instrument control, and the new Rapid Control product allows Active X control to be added to the Elite data system. This paper will describe these tools and give examples of their use.

### On-line analysis with a liquid sampling-atmospheric pressure glow discharge (LS-APGD)

*R. Kenneth Marcus and W. C. Davis, Clemson University, Department of Chemistry, 312 Hunter Laboratory, Clemson, SC 29634-0001, USA*

The successful implementation of any form of detection in microfluidic (gas or liquid) streams is a matter of both the obtained figures of merit as well as the compatibility of scale regarding the instrumentation and the concurrent capital costs. In the case of liquid sampling in the realm of capillary liquid chromatography or chip-based separations, the use of a multiple kilowatt plasma which operates in the 10 litres/min gas flow regimes (i.e. an inductively coupled plasma) does not make practical sense. In this laboratory, we have developed a liquid sampling-atmospheric pressure glow discharge (LS-APGD) optical emission source as a detector for flow injection or liquid chromatography separations. A low power is used though with sufficient energy to consume the eluting liquids totally at flow rates up to 0.5 ml/min. The components of the apparatus are depicted in the figure. The plasma operates in the abnormal glow discharge regime at currents of 30–80 mA and potentials of up to 2000 V. The LS-APGD is actually created such that the liquid sample acts as an electrode, in fact either as the anode or cathode. A simple sheath gas is employed in the current embodiment to alleviate pump-derived pulsation in the liquid flow when rates of ml injections. In absolute terms, this equates to about 10 ng analytes (Na, Mg, Hg, Pb) in a single injection. In this presentation, we will describe the operating characteristics of the LS-APGD with regards to various powering modes and its use as a detector in reverse-phase liquid chromatography. In addition to the metals listed above, the determination of halogen elements will also be described. Given the compact size, experimental simplicity and sensitivity observed to date, it appears that the device should have great promise as a detector for capillary LC or chip-based separations in the areas of environmental and potable water systems.

### A novel molecular aptamer beacon for real-time protein recognition

*Jinawei Jeffery Li[1], Weihong Tan[1] and Xiaohong Fang[2], [1]University of Florida, Department of Chemistry & The McKnight, PO Box 117200, Leigh Hal, Gainesville, FL 32611-7200, USA, [2]University of Florida, Institute of Chemistry, 52 Sanlihe Road, Beijing 100080, P. R. China*

One of the most pressing problems facing those attempting to understand the regulation of gene expression and translation is the necessity to monitor protein production in a variety of metabolic states. Yet, there is no easy solution that will either identify or quantitate proteins in real time. Recently molecular beacon based assays have shown promise for real-time protein detection. Here we introduce a novel approach to construct aptamer beacon for real-time protein recognition and quantitation. An aptamer beacon based on a thrombin-binding aptamer was prepared by labelling the two ends of the aptamer with a fluorophore and a quencher, respectively. The aptamer beacon combines the signal transduction mechanism of molecular beacons and the molecular recognition specificity of aptamers. Significant fluorescent signal change was observed when aptamer beacon was bound to thrombin, which is attributed to a significant conformational change in aptamer beacon from a loose random coil to a compact unimolecular quadruplex. The aptamer beacon recognizes its target protein with high specificity and high sensitivity in homogeneous solutions. Ratiometric imaging has been conducted with aptamer beacon labelled with two fluorophores, which makes it feasible for protein quantitation in living specimen. The unique properties of the aptamer beacon will enable the development of a class of protein probes for real-time protein tracing in living cells and for efficient biomedical diagnosis in homogeneous solutions.

### Rapid screening of environmental test wells by headspace mass spectrometry

*Brian G. Rohrback[1] and Elizabeth A. Harvey[2], [1]Infometrix, Inc., PO Box 1528, Woodinville, WA 98072-1528, USA, [2]Chevron Research, 100 Chevron Way, Richmond, CA 94801-2016, USA*

Gas chromatography is the preferred method for characterizing and quantitating low molecular weight compounds, but the separation process is often slow and often requires sample pretreatment. By eliminating the chromatograph and applying multivariate techniques to the bulk detector signal, composition can be determined quickly. Using a headspace sampling system with direct injection into a mass spectrometer, both qualitative and quantitative precision are demonstrated for a variety of environmental test applications. The short (3–5 min) analysis time and the lack of sample preparation create a fast screening technique for fugitive hydrocarbons. Although headspace technologies have gained some acceptance in the flavour and fragrance industry as a means of evaluating product and ingredient quality, little has been done to assess feedstocks, products or wastes in the petroleum industry. Data for this study are drawn from a set of 200+ test wells positioned at various locations within the boundaries of a refinery. For traditional quality assessment, classification techniques (such as nearest-neighbour and factor-based groupings) can be used to determine the contaminant origin even in cases where the candidate sources are chemically similar. Without resorting to GC separation, the concentration of classes of contaminants can be estimated over a four order-of-magnitude range.

### Personal Digital Assistant (PDA) LIMS: the integration of LIMS (Laboratory Information Management System) and mobile technology

*Russ Vranken, Don Kolva, Tom Miller and Christine Paszko, Accelerated Technology Laboratories, Inc., 496 Holly Grove School Road, West End, NC 27376-8412, USA*

As technology advances, more options become available for developers to create products that are both practical and functional for today's mobile generation. It has been proven in the past that automation is a vital key to the success of a Laboratory. The handheld devices of today are smaller and more powerful than they have ever been. For example, when Palm, Inc. first introduced the Pilot, it had a mere 512k of RAM which was good for the apps that were already integrated into the device, but limiting for additional applications. The PDAs of today have seemingly unlimited amounts of RAM (considering a Palm application typically takes up between 10 and 60k RAM) and a plethora of options making application development for these PDAs limited only by the imagination. One area of the LIMS that is in need of these types of applications is the sample login. With a LIMS application on the PDA, laboratory technicians in the field can easily enter the information about a collection session, return to the laboratory and simply 'hotsync' or upload to the LIMS with that information. Another function that will ease the burden of sample login will be to have pre-logged samples (samples waiting to be logged into the LIMS) sent to the PDA during synchronization and the laboratory technician in the field can simply enter the pertinent information related to the samples downloaded and synchronize back to the LIMS, automatically logging the samples in. In conclusion, the evolution of PDA technology and LIMS will allow laboratories to become more efficient in all aspects of sample and data management. As PDAs continue to advance, it is foreseeable that a full-featured LIMS could be implemented on these devices allowing laboratories to become even more mobile and efficient.

### Using software development methods for laboratory information management systems implementations

*Nicholas J. Arnold, Thermo LabSystems, 1 St George's Court, Altrincham WA14 5TP, UK*

As with many computer and other systems projects, the implementation of a Laboratory Information Management System (LIMS) can be a major undertaking for an organization. The success of a LIMS implementation project will depend on a number of factors including the technology employed, the composition of the project team and the involvement and buy in of the user community. Over the years, many software development methods have been designed or evolved, some of which can be used by project teams to facilitate their LIMS implementations. This paper will review a number of software development and project management methods that have been adapted for use in LIMS implementation projects. Particular emphasis will be placed on the use of the Dynamic Systems Development Method (DSDM), which is a Rapid Application Development (RAD)-based approach, several features of which have been employed by project teams on LIMS implementations in a variety of environments. Particular DSDM elements, which have been most successfully taken up and applied, include: user involvement, the use of facilitated workshops, high-level requirements, 80/20 thinking and prototyping The benefits of using such approaches will be discussed, along with the pitfalls. In addition, typical risks pertaining to LIMS projects, along with methods for assessing and mitigating them, will be presented.

### The role of diffusive sampling in the ambient air monitoring in Europe

*Richard H. Brown, Health and Safety Executive, Broad Lane, Sheffield S3 7HQ, UK*

Most early applications of diffusive sampling were for monitoring in industrial environments, where its simplicity and user friendliness gave it a positive advantage over sampling pumps. The EC Chemical Agents Directive, 98/24/EC, requires an assessment of a worker's potential exposure to chemicals as part of an assessment of any risk to his/her health and safety. This in turn generally requires measurements to be taken that need to be as cost-effective as possible to minimize any burden to industry. Thus, diffusive sampling is an ideal candidate. Similarly, the application of diffusive sampling techniques to ambient air has been given added impetus by European legislation, in this case the Ambient Air Directive 96/62/EC and its Daughters. These also require an assessment of air quality, by representative measurements, surveys or other means; it being assumed that measurements near the limit values signify a significant risk to the population or the environment. Depending on the anticipated concentration levels, different regimes and quality objectives for measurements are specified, and it is anticipated that diffusive sampling will be especially useful for 'indicative' measurements. The task of developing appropriate standards for workplace air quality measurements within the European Community has been carried forward by working groups of CEN Technical Committee TC 137. It took the view that air quality assessment standards should take the form of performance requirements rather than prescribed methods. This approach has the advantage of allowing any method to be used that meets these requirements without stifling innovation and development. Thus, CEN/TC137/WG2 has developed a hierarchy of standards with a general performance requirements document, EN 482, at the top and a series of specialized standards (e.g. EN 838 for diffusive sampling) under this umbrella. The parallel CEN committee for developing appropriate standards for ambient air quality measurements is TC 264. The primary task of this TC is to evaluate methods and recommend reference methods where these are not already prescribed in Directives. In the specific case of diffusive samplers, performance requirements standards (prEN 13528-1 and -2) have been developed by CEN/TC264/WG11. These draft standards are now being prepared for Final Vote and should be publicly available early in 2002.

### Advances in Membrane Extraction with Sorbent Interface (MESI) for on-site continuous air quality monitoring

*Janusz Pawliszyn, University of Waterloo, Department of Chemistry, 200 University Avenue West, Waterloo, Ontario N2L 3G1, Canada*

The ultimate goal of chemist is to perform analysis at a place where a sample is located rather than moving the sample to the laboratory, as is common practice in many cases at present. This approach eliminates errors and the time associated with sample transport and storage and it would result in more accurate, precise and faster analytical data. In addition to portability, two other important features of ideal field sample preparation technique are the elimination of solvent use and integration with a sampling step. These requirements are met by techniques using polymer technologies. Membrane Extraction with a Sorbent Interface (MESI) uses a polymeric membrane which is in contact with a sample fitted directly into a carrier gas line of a gas chromatograph equipped with a sorbent trap. Analytes partition into the polymeric phase of the membrane and after diffusion through the membrane are carried by the gas to the sorbent trap. Periodically the concentrated analytes are delivered onto the front of the column by a thermal pulse. MESI is a dynamic system where the rate of analyte intake is dependent on both the diffusion coefficients of analytes in the membrane material and the membrane/sample matrix distribution constant. Similar as SPME, MESI can be used for both spot- and time-averaged monitoring. Both SPME and MESI techniques integrate sampling with sample preparation and sample introduction to analytical instrument into a simple procedure. They are solvent-free techniques that not only facilitate convenient field sample preparation, but also rapid sample introduction resulting in fast chromatographic separations. In SPME, mechanical movement of the fibre is necessary; however, sampling and sample introduction steps are separated in space allowing one instrument to analyse large number of fibres. MESI on the other hand requires dedicated, permanently attached instrument to one or several membrane/sorbent systems, but it eliminates the need for mechanical movement and therefore reduces possibilities of failure. Fundamental aspects of TWA sampling by both techniques will be emphasized in the talk and their performance will be compared.

### Diffusion-based air sampling with solid-phase micro-extraction for indoor and ambient air

*Jacek Koziel[1], Jarett Spinhirne[2], Darren Williams[2] and David Parker[2], [1]Texas A&M University, Texas Agricultural Experiment St, 6500 W. Amarillo Blvd, Amarillo, TX 79106-1796, USA, [2]West Texas A&M University, Canyon, TX, USA*

Solid Phase Micro-Extraction (SPME) combines sampling and preconcentration. SPME uses extracting polymer coated on a fused silica fibre that is movable inside/outside of SPME needle assembly. Such a SPME configuration can be easily adapted to on-site air sampling and coupled with direct transfer of extracted analytes into a standard (or portable) gas chromatography (GC) system. Complete thermal desorption of analytes in a GC injector makes SPME fibres reusable. In the last decade, the theory of sampling with SPME was developed and applied to environmental samples including airborne volatile organic compounds (VOCs). These extractions are controlled by diffusion of VOCs onto the SPME coating, particularly when the SPME coating is significantly far from saturation. Diffusion-based calibrations are used for (1) time-weighted average (TWA) and (2) rapid sampling. For TWA sampling, SPME fibre coating remains retracted and analytes diffuse to fibre coating from the needle opening. The knowledge of gas-phase molecular diffusion coefficients (Dg), time ($t$), diffusion path ($Z$), needle opening area ($A$) and mass extracted ($n$) allows for VOC determination in air. SPME in the TWA sampling mode is applicable to long-term sampling. For rapid (spot) sampling, typically lasting < 1 min, VOCs diffuse through the boundary layer around the fibre coating that is exposed to forced air flow. VOC determination is based on the knowledge of Dg, $t$, $n$, length ($L$) and radius ($b$) of the SPME fibre, and the thickness of the boundary layer. This presentation will entail an overview of rapid and TWA SPME methods for determination of VOCs in air. New developments in rapid and long-term sampling will be illustrated with field data from indoor air surveys and agricultural odorous gases. Advantages and challenges associated with field air analysis with SPME will also be discussed.

### Selenium speciation by liquid chromatography: particle beam/hollow cathode optical emission spectroscopy: monitoring of carbon and hydrogen emission in selenoamino acids

*Fuxia Jin and R. Kenneth Marcus, Clemson University, Department of Chemistry, 312 Hunter Laboratory, Clemson, SC 29634-0001, USA*

As an essential trace mineral in the human body, selenium (Se) participates in anti-oxidative, cancer chemopreventative and immune-improving processes. Since the biochemical function of Se is highly species-dependent, the speciation of Se compounds rather than total Se determination is extremely important. Ion exchange and ion pair reverse-phase HPLC mainly have been applied to the separation of Se species. An ion pair reverse-phase HPLC method for the separation of selenomethionine, selenoethionine and selenocystine was optimized to have a mobile phase composition of 95% water–5% methanol containing 0.1% TFA [1]. A diversity of methods was employed for the detection of Se content, which includes electrochemistry, UV detection, fluorometry, atomic spectroscopy and mass spectrometry. We have successfully developed a new liquid chromatography method for the separation and speciation of organic and inorganic Se compounds via liquid chromatography/particle beam hollow cathode optical emission spectroscopy system (LC/PB-HCOES) [2]. A detailed systematic study of selenoamino acids separation on four commercial reverse-phase C18 columns by reverse-phase chromatography has been performed. The baseline resolved separation of selenoamino acids by reverse-phase liquid chromatography with a high methanol content up to 15% in the mobile phase without the presence of any ion pair agents has been achieved. This greatly facilitates the sample preparation procedure and favourably enhances the feasibility of interfacing liquid chromatography to particle beam system. The successful separation of selenoamino acids with a mobile phase of only water and methanol makes it possible to identify the selenoamino acids through the analysis of carbon and hydrogen elements. The carbon and hydrogen optical emission in the selenoamino acids was monitored for the LC eluents after separation. The effects of solvent composition and glow discharge conditions on spectroscopic background were studied. The analytical response curve for integrated C and H emission intensities as a function of the concentration was obtained.

1. Puskel, E., Mester, Z. and Fodor, P. J. *Analytical and Atomic Spectrometry*, 14 (1999), 973.
2. Davis, W. C., Jin, F., Dempster, M. A., Robichaud, J. L.. and Marcus, R. K. J. *Analytical and Atomic Spectrometry* (in press).

### Method 1631: mercury holding time study

*Mark L. Bruce, Scott Irwin and Patrick Omeara, Severn Trent Services, STL North Canton, 4101 Shuffel Drive NW, North Canton, OH, 44720-6935, USA*

US EPA Method 1631C is required or recommended for total mercury analysis in many types of water matrices in the low parts per trillion concentration range. Currently, the method requires that the water sample be preserved with either HCl or BrCl within 48 h of sample collection. Preservation in the field involves the shipment of hazardous chemicals and additional manipulations that increase the risk of accidental contamination. Thus, preservation at the laboratory is preferred. To accomplish laboratory preservation currently necessitates overnight shipment on the day of collection and day of receipt preservation at the receiving laboratory. This rapid action in the field and in the laboratory is possible most of the time but there are numerous instances where this rapid sample handling is impractical, expensive and, in some cases, impossible. Lengthening the unpreserved holding time would facilitate more efficient sample handling both in the field and at the laboratory. A holding time study was undertaken to demonstrate that total mercury content in water sample is stable for much longer than 48 h. Four different types of water samples (reagent water, freshwater pond, industrial effluent and publicly owned treatment works effluent) with concentrations between 1 and 5 ng/l were studied over 35 days. Aliquots of each sample were held unpreserved for 7, 14, 21, 28 and 35 days. The samples were then analysed by Method 1631. Statistical comparisons of the linear least-squares regression line of each sample data set indicates there was no significant change in concentration during the 35 days of the study. Thus, the unpreserved sample holding time for total mercury as measured by Method 1631 should be extended to at least 28 days provided the sample is oxidized in the original sample collection container. The effluent from a publicly owned treatment works shows stability of the unpreserved sample over a 35-day test period, the variation being not significantly different from zero at 0.01 ng/l/day.

### The direct mercury analysis: a revolutionary approach in mercury analysis

*Mikhail Mensh, Milestone, Inc., 160B Shelton Road, Monroe, CT 06468-2545, USA*

For many years, the bottleneck of mercury analysis was sample preparation. To perform analysis of a sample using one of the traditional methods such as cold vapour, the sample has to be properly collected, stored and processed (digested and chemically treated). Those steps are the most time, labour and money consuming. The final analysis step takes only a few minutes per sample. Direct analysis of mercury using a Direct Mercury Analyzer (DMA-80) affords many benefits. Eliminating wet chemistry greatly reduces waste generation, systematic errors and technician exposure due to volatilization of mercury during sample handling. Direct analysis typically provides an answer in approximately 5 min after a sample has been introduced into the instrument. A wide dynamic range, from 0.5 to 600 ng total mercury in a sample, is easily accommodated. Configuring the instrument for deployment in the field can reduce systematic errors due to improper sample storage. Direct mercury analysis in the field, near remediation and clean-up activities can result in significant improvements in the accuracy of sample data. On-site analysis can significantly impact the dynamics, as well as the economics, of remediation activities by providing timely information for site management. DMA-80 uses the integrated sequence of Thermal Decomposition, Catalytic Conversion, Amalgamation and Atomic Absorption Spectrophotometry. It is described in EPA Method 7473 and is validated for laboratory and field analysis.

### Validation of EPA Draft Method 3200: mercury speciation by selective solvent extraction and acid digestion

*Mizanur Rahman[1], Ye Han[1], Helen M. Boylan[1], Sejal Shah[2] and H. M. Kingston[3], [1]Duquesne University, Chemistry Department, 600 Forbes Avenue, Pittsburgh, PA 15282-0001, USA, [2]Duquesne University, Department of Chemistry and Biochemistry, 308 Mellon Hall, Pittsburgh, PA 15282-0001, USA, [3]Duquesne University, 1225 E. Arques Avenue, Sunnyvale, CA 94085-4701, USA*

The need for speciation analysis, i.e. the determination of specific chemical forms of an element, arises from the necessity of determining the concentration of those species, which are characterized by the highest toxicity, environmental mobility and tendency to accumulate in living systems. Mercury species can undergo a variety of transformations in the environment owing to the interconversion and degradation processes, especially for complex solid matrices such as soils, sediments and biological tissues. One of the most important processes is methylation. Investigations showed that such methylation process mainly observed in samples rich in organic matter and high in inorganic mercury. This transformation can also occur during extraction and acid digestion processes. The extraction of mercury and mercury compounds from environmental, biological and botanical sample is very complex. The extraction should be performed in such a way that the analyte is separated from the interfering matrix without loss, contamination or change of the speciation, and with minimum or no interference. A draft Method 3200, Mercury Species by Selective Solvent Extraction and Acid Digestion, has previously been developed to extract selectively ‘mobile and toxic’ species such as alkylmercury, soluble inorganic mercury from soils and sediments. This study mainly focuses on the validation of Method 3200 using standard soil samples. High-performance liquid chromatography (HPLC) is coupled with the most sensitive elemental detection method inductively coupled plasma mass spectrometry (ICP-MS) to perform mercury speciation studies. Method 7473, a rapid technique for the determination of total mercury in environmental and biological samples, is used to compare results obtained form HPLC-ICP-MS. Interlaboratory data demonstrating method effectiveness are also presented and evaluated.

### Ageing effects on mercury speciation in soil samples

*Manuel Valiente and Xavier Gaona, Universitat Autonoma de Barcelona, Department de Quimica, Edifici CN, Bellaterra, Barcelona E-08193, Spain*

Because of the different bioavailability and toxicity of inorganic and organo-metallic species of mercury that can be present in metal-contaminated sites, a corresponding speciation procedure for the proper determine of the content of the different metal species must be applied. For soil samples, the most accepted procedure includes the solvent extraction of organo-mercury species such as methyl-mercury (MeHg) and phenyl-mercury (PhHg) among others. Besides volatilization, one of the key process that takes place in this system is the degradation of organo-mercury species with time. Ageing effect will account for both the corresponding loss and transformation of organo-mercury species. The present work is dedicated to ascertain some steps of the sample treatment and analytical procedure that influences this ageing effect. For organo-mercury determination, we used the capillary electrophoresis technique provided with a UV detector. We identified three steps that influence the ageing effect: sampling, preparation and conservation of standards solutions, and conditioning and storing of the soil sample extract. The results, obtained by monitoring MeHg and PhHg in soil samples, show a clear difference on the behaviour of both species. Thus, the ageing effect for MeHg is different than for PhHg in the sampling procedure (use or absence of sample freezing). On the other hand, the use of cysteine as a complexing agent in both sample treatment and preparation of standard solutions influences the ageing effect in a different manner. Thus, the standard calibration curve has a better repetitivity when cysteine is added to the organo-mercury standard just before the CE measurement. The ageing effect is also observed on the resolution of the CE what is attributed to cysteine degradation. The illustration of the results will follow the presentation of the experimental methodology employed in this study.

### The application of a new separation technology to the conditioning of wastewater samples for on-line analysers

*David Griffiths, Filtra Ltd, Biomedical Building—FL1000, Australian Technology Park, Ste G11, Eveleigh, NSW 1430, Australia*

The use of sophisticated on-line analysers in wastewater treatment plants can lead to increased efficiency and improved environmental outcomes. Unfortunately, the difficulties of sampling the process liquors have inhibited the use of such analysers in the wastewater and other industries. In particular, the need to separate out a range of often sticky, fibrous and generally difficult solids without using maintenance-intensive equipment has been an issue for the industry. Filtra Ltd, an Australian start-up company, has developed a new solids/liquid separation technology that has particular application to the pretreatment or conditioning of wastewater samples for sophisticated on-line process analysers. The technology provides a reliable sample that can be piped significant distances with minimal sample degradation before analysis. Unlike traditional conditioning devices, the new technology is intrinsically non-blocking and has minimal maintenance requirements. Case histories from both pilot plant and operational facilities are presented. The application of the technology is demonstrated in a variety of situations including fast-loop systems and in multiplexed arrangements where multiple sample sources are analysed using a common analyser.

### NoteBookMaker is a completely electronic, totally secure laboratory notebook

*Lance Boynton and Stephen Arpie, NoteBookMaker, PO Box 5585, Hamden, CT 06518-0585, USA*

NoteBookMaker™ is a completely electronic, totally secure laboratory notebook. In the pharmaceutical industry, the ability to control and easily access securely all information pertinent to a specific project or a series of projects is essential. The capability of performing a quick search can greatly increase efficiency and therefore reduce the cost of a laboratory. In the current paper format, to search for relevant information, a scientist must locate the correct serialized notebook and then manually find the correct reference in that publication. With NoteBookMaker, a simple electronic search can find all the related topics in a matter of seconds. Even with the easy-to-use graphical user, interface security is still held at a premium. In fact, NoteBookMaker meets all the requirements for an Electronic Notebook established by CENSA (Collaborative Electronic Notebook Systems Association), a consortium group that includes individuals from several disciplines including end users, government and industry. NoteBookMaker creates legally defensible data and is compliant with 21 CFR Part 11. The first real alternative to paper-based systems, it is extremely easy to use and a breeze for IT professionals to manage. Pages are created on demand and look and print like genuine pieces of paper. A program that enhances the communication between chemists and administrators while maintaining the highest level of customizable security is fundamental in today's competitive marketplace.

### Rapid on-site analysis of environmental soil samples in a mobile laboratory using the pressurized solvent extraction technique

*Al Kaziunas[1], Rolf Schlake[1], Mark Mathews[2] and Michael Winslow[2], [1]Applied Separations, 930 W. Hamilton Street, Allentown, PA 18101-1114, USA, [2]KB Labs, 6821 SW Archer Road, Gainesville, FL 32608-4720, USA*

The need for rapid analyses of environmental soil samples requires mobile laboratories to extract and analyse samples on site. Unfortunately, the goal of providing analytical results in the same day is hindered by time consuming, traditional solvent extraction techniques. This paper investigates the use of the pressurized solvent extraction technique (EPA Method 3545) to reduce the time required to extract soil samples in a mobile laboratory. Soil samples were extracted on a compact pressurized solvent extraction system and analysed for organochlorine pesticides, PCBs and PAHs using SW846 methods 3031A, 8082, and 8100. Results were compared with typical off-site laboratory analyses. In addition, a calculation of timesavings and reduction of solvent usage is presented.

### Portable environmental chemical sampler

*Jim Smith and Ron Skinner, Prince Edward Island Food Technology Centre, PO Box 2000, Charlottetown, PE C1A 7N8, Canada*

A prototype portable environmental chemical sampler using Solid-Phase Micro-Extraction (SPME) has been manufactured and evaluated for environmental applications. Its small size and the stability of sampled environmental chemicals on the SPME fibres makes the device suitable for many environmental applications including the sampling of pesticide run-off into rivers, pesticide spray drift and effluent monitoring. A carousel of SMPE fibres is contained within the sampler and each fibre is exposed to the environment to be sampled. The sampling sequence can be initiated by various sensors, cellphone modem or preprogrammed into the sampler microprocessor. A typical sampler application would be to sample river water using a carousel of 48 SPME fibres over a 48 h with each fibre being exposed for 1 h. A separate sentinel fibre in the sampler is exposed for the whole 48 h. Once the environmental chemicals are adsorbed onto the fibres, they are very stable, unlike liquid samples taken in bottles. There is therefore no urgency to return the samples to the laboratory for analysis. When the SPME fibres are returned to the laboratory for analysis, the sentinel fibre is analysed first. If it is clean, the carousel does not need to be analysed, saving on analysis costs. If the sentinel fibre is positive for the contaminant of interest, the carousel of fibres is placed into the modified GC autosampler and the fibres are sampled automatically. Field trial evaluations have been conducted and will be presented in detail.

### Process control through real-time plasma monitoring for endpoint, fault and state-of-health

*Pamela P. Ward, Peak Sensor Systems, Research and Development, 6207 Pan American NE, Albuquerque, NM 87109-3425, USA*

Plasma processes used for production purposes are dynamic environments in which minute changes in plasma chemistry can have devastating effects on process outcome. In today's competitive environment, manufactures of semiconductors, printed circuit board or other high dollar products in which plasma is a critical production step can not tolerate processes that are not controlled. This paper addresses a real-time, full-spectrum optical plasma-monitoring technique capable of monitoring all plasma processes, recipes and products for endpoint, occurrence of faults and the overall general state of health of the plasma environment. Data will be shown that will demonstrate the increase of throughput, yield and overall quality of products monitored via this technique. Through the application of the ProPak in the semiconductor industry in a three year study, results have indicated that the end user can expect increases to throughput as high as 35%, yield increases as high as 12%, and better device performance and clock speeds. Furthermore, a list of captured fault conditions that have prevented the loss of millions of dollars of products will be shared along with a methodology to detect and prevent any plasma fault that has a distinguishable optical indicator. In this discussion, the operation of the ProPak plasma monitor on AMAT, LAM and TEL reactors, and the overall benefit to the semiconductor industry will be shown. Data from real-world production environments will be shared to demonstrate the benefit of plasma monitoring. Advancements in automatic process control and sophisticated communication techniques will be discussed.

### High-throughput screening of polyolefins made using single site catalysts

*Judy Arroyave[1] and Wei Sen Wong[2], [1]Albemarle, R&D, 8000 GSRI Road, Box 14799, Baton Rouge, LA 70820-7403, USA, [2]Albemarle, PO Box 14799, Baton Rouge, LA 70898-4799, USA*

As a leading manufacturer of methylaluminoxanes (MAOs), metallocene catalysts and other catalysts components, Albemarle Corporation has a key interest in characterizing their performance in a wide variety of polymerization reactions. To facilitate this research, Albemarle has developed a high-throughput screening approach to incorporate the polymerization reactions, isolation and characterization of these product materials. As part of this effort, it became necessary to develop a rapid GPC technique that would provide accurate data and faster analysis time while maintaining data integrity. This presentation will give an overview of Albemarle's high-throughput screening programme related to catalyst performance. It describes a GPC technique that allows the screening of these reactions, determining critical information such as weight-average molecular weight, intrinsic viscosity and radius of gyration. Laboratory automation along with a unique combination of solvent and specialized columns have yielded very favourable analysis conditions while maintaining an excellent degree of accuracy and reproducibility.

### Combinatorial screening of polymer libraries using rapid chromatographic and related techniques

*Miroslav Petro, Symyx Technologies, Inc., 3100 Central Expy, Santa Clara, CA 95051-0801, USA*

The combinatorial chemistry approaches supported by rapid automated analytical techniques lead to a significant acceleration of discovery process in several different areas, including material polymer science. At Symyx, we have developed a variety of techniques and approaches based on interaction, separation, molecular recognition and detection of macromolecules in solution. Those techniques have been successfully applied to a number of different material discovery projects either to identify or to characterize rapidly the best polymer candidates from our libraries of materials. The presentation will give an overview of methodologies for rapid determination or assessment of the molecular size, molecular architecture, chemical composition, multiple distribution profiles of molecular characteristics, interaction and reaction capabilities of macromolecules at a speed that is satisfactory to match the speed of parallel polymer synthesis. The techniques are being applied for a large diversity of polymers ranging from those soluble only in organic solvents at high temperatures to those requiring aqueous or ionic environment. In addition, several aspects related to development of these methodologies and their application for identification of hits in our polymer discovery programmes will be discussed.

### Automated methods for characterizing equilibrium properties and non-equilibrium properties in polymer solutions

*Wayne F. Reed, Tulane University, Physics Department, Stern Hall, New Orleans, LA 70118, USA*

Automatic, continuous mixing techniques (ACM) can be used to characterize a vast number of equilibrium and non-equilibrium characteristics of polymer solutions. Using ACM with combinations of light scattering, viscometric, ultraviolet absorption and refractometric detectors, without using any chromatographic columns, examples of the following will be given. (1) Automatic on-line monitoring of polymerization reactions (ACOMP), which furnishes molecular mass, monomer conversion and other characteristics. Applications of ACOMP to free radical, controlled radical and step growth reactions will be discussed. Recently adapted to copolymerization reactions, ACOMP allows simultaneous, on-line determination of copolymer composition distributions, sequence lengths, mass distributions and reactivity ratios. (2) Determination of kinetics and mechanisms involved in polymer degradation, which also yields information on polymer structure, such as branching and multiple stranding. (3) Monitoring of solution instability in the form of aggregation, phase separation and micro-crystallization. ACM is also readily adaptable to monitoring the dissolution of dry polymers. In addition, ACM methods have been adapted to understand complex interactions in multicomponent systems, such as those containing polymer and surfactants, and polyelectrolytes and salts. Finally, new instrumentation for massive parallel testing and high-throughput screening will be mentioned.

### Rapid screening of drugs for forensic toxicology using retention time locking with gas chromatography/mass spectrometry

*Michael Szelewski[1], Fran Diamond[2] and Paul Miller[3], [1]Agilent Technologies, 2850 Centerville Road, Wilmington, DE* 19808-1610, USA, *[2]National Medical Services, 3701 Welsh Road, Willow Grove, PA* 19090-2910, USA, *[3]National Medical Services, 2100 Clearwater Drive, Des Plaines, IL, 60018-1918, USA*

Identification of drugs in a biological matrix is challenging due to interferences and analyte degradation. A typical analysis involves a library search of all peaks found in a GC/MS run followed by a manual confirmation by a trained analyst. Retention time locking allows exact retention times on one instrument or across multiple instruments. These exact retention times can be reproduced after column maintenance, which is a daily procedure in many laboratories. A retention time locked database of 300 drugs and anayltes of interest to forensic toxicologists has been developed. Chromatographic peaks are searched against the database first for retention time and then for target and qualifier ion ratios. A cross correlation against the library spectrum is also performed automatically. Combining retention time with spectral information reduces the number of false-positives and -negatives. This rapid screening process for 300 analytes is completed in < 1 min after the chromatographic run.

### Achieving new levels of reliability in automated chromatography

*James A. Schibler[1], Martina Oefelein[1] and Andreas Becker[2], [1]Dionex Corporation, 1228 Titan Way, Sunnyvale, CA 94085-4074, USA, [2]Dionex Corporation, Via G. Fantoli 16/15, Milan I-29138, Italy*

Most modern chromatography laboratories use software to automate instrument control and data processing in order to minimize the operator time required per analysis. Although automation can substantially increase productivity when everything is working well, most automated systems are unable to respond effectively when hardware or method problems occur. Common problems that afflict automated systems include power outages, computer-related problems (such as network communication failures or data storage problems), instrument component failures and inadequate chromatographic performance (peak tailing, coelution, poor precision, etc.). Whether the problem causes the system to halt prematurely or to continue processing samples without generating usable data, the consequences are costly: valuable samples and reagents can be wasted, and time—the most precious resource of all—is lost. This presentation will discuss how a modern chromatography management system intelligently and automatically handles such contingencies, thus providing new levels of reliability and productivity improvement in automated chromatography.

### Monitoring with MESI

*Heather Lord[1], Limei Wang[1], Rick Morehead[2] and Janusz Pawliszyn[3], [1]University of Waterloo, Chemistry, 200 University Avenue West, Waterloo, Ontario N2L 3G1, Canada, [2]Restek Corporation, 110 Benner CIR, Bellefonte, PA 16823-8497, USA, [3]University of Waterloo, 29 Business Park Drive, Branford, CT 06405-2967, USA*

Membrane Extraction with Sorbent Interface (MESI) technology has been used for continuous monitoring of forensic, environmental and fragrance samples. It has been used for both spot sampling of air concentrations of chemicals as well as integrative sampling to assess time-weighted average concentration. The feasibility of MESI for breath sampling was assessed with the analysis of breath exhaled while smoking, and with the analysis of breath alcohol after consuming an alcoholic beverage. End tidal breath samples were collected and analysed in a sampler designed to trap the last 250 ml of breath. Early breath samples were collected by exhaling into a sampling jar. PDMS membrane and a DVB trap and 10-min trapping times were used for all samples with breath sample analysis by GC/FID. Several significant additional peaks were observed in the breath of a smoker during smoking, as opposed to just before smoking. Ethanol was easily observed in the breath after alcohol consumption, and the rate of disappearance was monitored. Freshly fallen snow was analysed for the presence of BTEX compounds adsorbed during snowflake formation. Peaks corresponding to benzene, toluene and xylenes were observed with signal-to-noise ratios of 3:1 to 10:1 by GC/MS analysis from the headspace of 300 ml of melted snow. Finally, eucalyptus volatiles were monitored from both fresh and day old leaves. All of the major monoterpene components, as well as some of the lighter sesqui-terpenes and green leaf volatiles, were observed. Variation of eucalyptus volatiles could be monitored relative to both maturity and senescence states of the leaves.

### Improving Raman shift measurement using automatic simultaneous Raman and laser calibration

*Jun Zhao and Mike M. Carrabba, Chromex, Inc., 2705 Pan American Fwy NE, Albuquerque, NM 87107-1630*

Accurate and precise measurement of Raman shift value is of paramount importance to Raman spectroscopy, particularly for demanding in-line monitoring applications. Since the Raman abscissa is a shift relative to the laser frequency, both the Raman and the laser frequency must be known. The wide spread use of compact semiconductor lasers and solid state lasers has compounded the problem due to the frequency instability of the excitation source. Unfortunately, conventional disperse Raman spectrometers are not equipped to characterize the excitation source. A new generation of disperse Raman instrument was developed to cope with this difficulty by simultaneously measuring the Raman and the laser spectra and calibrating them by the means of accurately known atomic emission lines. A single CCD detector attached to a high-quality imaging spectrograph collected all the data. In this study, a 532-nm frequency-doubled Nd:YAG laser from a well-known vendor was used and its frequency monitored. The inherent frequency instability of the laser caused large systematic errors in Raman shift if it was uncounted for. The new instrument characterized the laser frequency for each Raman measurement and yielded consistent results. Precision was improved by an order of magnitude to $> 0.05\,cm^{-1}$. Such reliable measurement will benefit Raman spectroscopy for industrial process applications.

### Automated interpretation of multivariate instrument data

*Marlana Blackburn, Scott Ramos and Brian Rohrback, Infometrix, Inc., PO Box 1528, Woodinville, WA 98072-1528, USA*

Several commercial packages for chemometric data analysis are currently available, including Pirouette. InStep, a free companion programme, is a custom client communicating directly with Pirouette. It automates multivariate prediction and allows the user to customize easily an analysis scheme and report format. In its processing mode, InStep periodically checks a watched folder for a file of specified extension, e.g. a type produced by a spectroscopic or chromatographic data acquisition system. When a new file appears, predictions are made on its data using an InStep method. Tabular results can be displayed on screen and/or written to a file. Control charting of predicted values is also provided. In its set-up mode, the user can create methods and design reports. An Instep method is a recipe for processing each sample. The simplest method consists of one model; however, additional processing can be triggered by testing results of the initial prediction and branching conditionally. For example, if the predicted Y2 from Model A is greater than a user supplied value, apply Model B, otherwise apply Model C. The comparisons and branching can continue indefinitely.

### Optimizing purge-and-trap/mass spectrometry systems for high performance

*Cameron George[1] and Philip L. Wylie[1,2], [1]Agilent Technologies, Technical Support, 91 Blue Ravine Road, Folsom, CA 95630-4720, USA, [2]Allen K Analytical Sciences, Five Moore Drive, RTP, NC 27709, USA*

The analysis of volatile organic compounds (VOC) in various matrices is a primary concern for most environmental testing laboratories. Recent changes in MS detector design have resulted in improved sensitivity allowing for potential method improvements. With these improvements has come the need for optimizing all system parameters, including purge-and-trap conditions, column choice, detector settings and tune values. This paper discusses the effects of these changes on sensitivity, linearity, analyte resolution and analysis time. An optimized set of conditions for the analysis of EPA Method 8260B will be considered in detail and all instrument conditions will be detailed.

### Improvements on blood alcohol content determination using autosampler vial headspace sampling coupled with gas chromatographic analysis

*Charles C. Johnson, Martin Paplewski and John W. Hellgeth, JAS, Inc., 4 Highway Avenue, Ludlow, KY 41016-1663, USA*

With the Federal initiatives for highway funding being dependent upon State compliance with a 0.08% blood alcohol limit, police and forensic laboratories are under increased pressure to provide rapid blood alcohol analyses. Classical analyses using conventional headspace samplers have intrinsic limitations on sample throughput. Previously, a valid method under California Title 17 legislation was reported called the Blood Alcohol Reporting System (BARS). This method demonstrated the accuracy and precision necessary to comply with the Title 17 criteria can be accomplished using the headspace of a sample within a 2-ml Autosampler Vial. Improvements to the BARS method, both in the procedure and data analysis, will be presented.

### Chaotic analysis of electrochemical noise response of copper

*Esteban M. G. Ochoa[1], Edgar A. C. Ibanez[1] and Guillermo A. V. Coutinho[2], [1]Instituto Mexicano del Petroleo, Production Management, EJE Central 152, Mexico Distrito Federal, Distrito F 07730, Mexico, [2]Instituto Mexicano del Petroleo, Iztapalapa, Mexico*

The aim of the present study has been to analyse the current oscillations during the pitting corrosion process of copper, joining the information offered by traditional techniques of electrochemistry (voltammetry), such as reverse-scan polarization, with the analysis of time series of the non-linear current oscillations under potentiostatic conditions during the corrosion process. Furthermore, the parameters obtained from the application of the chaos theory allow us to determine whether or not the phenomenon is of chaotic nature, and to assess the number of necessary differential equations to describe the system as well. Besides, we can say that the dynamic changes in function of different species. Corrosion produced by pits has different reactions in each step with its own strange attractor. Recent developments in the theory of linear dynamics have demonstrated the existence of very complex solutions to certain very simple differential equations. These solutions exhibit apparently random fluctuations. In the case of dissipative systems, the solutions curves eventually remain confined to a subset of the phase space. This subset is known as an attractor. Chaotic behaviour occurs when a stranger exists.

### Automation of *in vitro* drug liberation using flow methods

*Petr Solich*[1] *and Uli Schaefer*[2], [1]*Charles University, Faculty of Pharmacy, Analytical Chemistry, Heyrovskeho 1203, Hradec Kralove, 500 05, Czech Republic,* [2]*Saarland University, Biopharmaceutics and Pharmaceuti, Saarbruecken D-66123, Germany*

One of the most widely accepted standard for monitoring of semisolid pharmaceuticals is to determine the rate of release of active pharmaceutical ingredients with respect to the time. Recently, according to the recommendation of FDA, the use of the Franz cell system for liberation of topical preparations is recommended. The studies should provide the multipoint-profile testing, which commonly can be done by HPLC analysis (usually with UV detection). Although widely used, this method is time-consuming, with a low frequency of sampling. Therefore, a new methodology based on flow methods FIA and SIA is recommended for testing of *in vitro* drug liberation from topical pharmaceuticals. Flow analytical methods (flow injection analysis (FIA) and sequential injection analysis (SIA)) are predicted for the automation of analytical procedures. The interfacing of computer-aided FIA or fully automated SIA with an sensitive fluorescence detector and Franz cell leads to a fully automated monitoring system capable of providing real-time analysis and a multipoint liberation profile. Examples of liberation profiles for a topical dermatological formulation containing the active compound salicylic acid are shown. The use of fluorimetric detector increases the selectivity and sensitivity in comparison with the conventional UV detection used in HPLC. The recommended time for the analysis of the liberation profile is 6 h, using sampling at 0.5, 1, 2, 4 and 6 h after beginning of the procedure. We have found that the more frequent sampling—every 2–6 min, which can be done by using and automated FIA or SIA system—enables one to construct a straight line (release rate) after only about 3 h instead of the usually used 6 h. Therefore, it can be concluded that using the FIA procedure with the possibility of analysing 10–30 samples per hour, the release rate can be calculated after about half time of the usual measurement time. This example shows the attractivity and potentiality of automated flow methods FIA and SIA for automated monitoring of liberation of active drug substances from topical applied dermatological formulations.

### Rapid detection of genetic variations using an automated capillary electrophoresis system

*Dan Zhu*[1,2], [1]*Beckman Coulter, Inc., Life Science Research Division, 4300 N. Harbor Blvd, Fullerton, CA 92835-1091, USA,* [2]*Beckman Coulter, Inc., Little Falls Site, 2850 Centerville Road, Wilmington, DE 19808-1610, USA*

Capillary electrophoresis (CE) has had a major impact on genomics with its significantly increased productivity over slab gel electrophoresis in DNA sequencing. As increasingly more DNA sequencing data become available, more studies are required to understand better the association between genomic abnormalities and diseases as well as the significance of the DNA variations among individuals. Single base pair mutations, deletions and insertions of specific genes are known to cause specific diseases while single nucleotide polymorphisms (SNPs) may lead to individual differences between disease susceptibility and response to treatment. We have developed a non-sequencing approach to detection of mutations/SNPs in a high-throughput and cost-effective manner using an automated multifunctional CE system. The approach is based on the fact that, under certain temperature (range), DNA variant with as little as single base pair difference from a wild-type sequence itself and the heteroduplexes formed between the variant and the wild-type may migrate at different rates compared with the wild-type sequence in a denaturing polyacrylamide gel. In this study, we used this approach to detect mutations at codons 12 and 13 of K-ras gene that is mutated in a variety of human cancers. DNA samples with all the possible mutation types at the first base sites of the two codons were tested and all the mutations were detected without ambiguity. The results demonstrate that our non-sequencing CE-based approach significantly speeds up mutation/SNP detection processes with accuracy no less than that of sequencing and primer extension approaches.

### An automatic information system and its use in voltammetric analysis

*Rashida M.-F. Salikhdzhanova*[1,2], *Alexei I. Gorobets*[1], *Natalia J. Petrova*[1] *and Damir I. Davletchin*[3], [1]*Moscow Institute of Radio Engineering and Electronics, Radio Constructioning, Lenin's Square 1 80, Moscow 113114, Russia,* [2]*Moscow Institute of Radio Engineering and Electronics, Analytical Research & Development, Experimental Station E353/3, Wilmington, DE 19880-0353, Russia,* [3]*Moscow Textile Academy, Chemistry Department, Moscow, Russia*

Voltammetric analysis is based on individual methods for every object determination that define operating conditions for the voltammetric instrumentation. These methods give recommendations on the choice of supporting electrolytes, the electrode system, the potential range for the analysed substance and the voltammetric technique to be used. A system of expertise for compiling an automated data library for voltammetric methods has been presented. This data library is a database system for automatic retrieval of information that makes use of selection criteria such as the objects of determination criteria such as the objects of determination and analysis, and background. The system can be used for crating specialized databases of voltammetric methods for use in environmental monitoring, industry and medicine. Data include descriptions of some 1000 voltammetric methods. The information system includes a descriptive presentation of methods in complete form as reported in the literature and in a concise tabulated form. Information, presented in tabular form, is retrieved automatically from the library using the following criteria: object of determination, object of analysis, background, indicator electrode and type of voltammetric method.

### Automation of Karl Fischer methods for the determination of water content

*Stephanie P. Wilson[1], Lynn Jordan[1], Terry Roche[2] and Elise Corrigan[2], [1]Zymark Corporation, Zymark Ctr, Hopkinton, MA 01748-1668, USA, [2]Zymark Ltd, Systems Engineering, 1 Wellfield Preston Brook, Runcorn, WA7 3AZ, UK*

In 1935, Karl Fischer published information on the determination of water content, which has become the global standard over many industries. Automation of the Karl Fischer process has been minimal. With the integration of a standard Karl Fisher volumetric titrator, using disposable sample holders, and integration with robotics, automation of this labour-intensive task can result in higher throughput, improved reproducibility, cost savings and promote standardization across industries including those in the regulatory environment who must meet compliance to 21 CFR Part 11. The authors will provide information on the process of automating the Karl Fischer determination of water content through the use of current technology and integration of these technologies.

### Electronic nose as a quality control tool in packaging industry

*Tsung Tan[1], Nicolas Mignard[1], Quitterie Lucas[1], Helge Nickel[2] and Alexander Stofell[2], [1]Alpha M.O.S., 20 Avenue Didier Daurat, Toulouse F-31400, France, [2]Basell Polyolefine GmbH, Industriepark Hoechst Build, Frankfurt D-65926, Germany*

The consistent manufacture of high-quality PE pellets for drinking water applications is extremely important for this industry in general and for Basell GmbH. Traditional methods for taste tests require soaking pellets in water and conditioning them to carry out the tests. Then serial dilutions are made until no odour or taste are found. These tests are time-consuming, require a minimum number of sensory panellists and are very repetitive. Using these samples, the Fox Electronic Nose was used to obtain results correlated with these tests. After validation, the system has been implemented in a QC environment. The Fox Electronic Nose has now been used in QC laboratory for over 2 years. Reliable and objective results have been obtained in a production environment where the tests are completed four times faster than a standard QC sensory panel. These results will be presented with emphasis of the reliability of such instruments over the long-term, the effect of sensor replacement and also transfer from R&D to QC. New results will also be shown on a lower cost, high-throughput electronic nose—GEMINI. The instrument can provide results every 5 min in a cost-effective package with high reliability.

### Statistical quality control of petrochemical and oleochemical products using the new factory compatible electronic nose

*Quitterie Lucas, Vincent O. Schmitt and Tsung Tan, Alpha M.O.S., 20 Avenue Didier Daurat, Toulouse F-31400, France*

Detection of tainted products due to off-odours and off-tastes in incoming materials and final products is essential for chemical and packaging industries to assess product integrity and meet customer acceptance especially for food and pharmaceutical applications. The increasing number of controls by factory human panels have led to the development of a fast, accurate and high-quality electronic nose for comparison of manufactured product with ‘gold references’. A description of a powerful 18-sensor FOX 4000 Electronic Nose will be presented to illustrate both quantitative and qualitative applications. The final results will then be presented on an optimized six-sensor GEMINI system. Several applications in the polymer and petrochemistry with the GEMINI Electronic Nose assess product integrity and provide a statistical quality/process control (SQC/SPC) chart in production facilities. The performances of the system will be addressed in terms of accuracy, reproducibility and sample throughput.

### Data acquisition and analysis software for gel permeation chromatography with single or multi-detector capabilities

*Stephen O’Donohue, Elizabeth Meehan, Geoff Cowell and Greg Saunders, Polymer Laboratories Ltd, Essex Road, Church Stretton SY6 6AX, UK*

In contrast to regular HPLC applications, the software requirements for data analysis in gel permeation chromatography (GPC) are very specific. In the simplest case, using a single concentration detector, well-established algorithms can be applied. Additional detectors add complexity to the data analysis and any GPC-specific package must be flexible enough to address a variety of multidetector applications. This presentation reviews the philosophy of an approach to addressing these requirements in a new GPC software suite, Cirrus™. Mathematical functionality is obviously a prerequisite and the capabilities of single and multidetector software modules will be illustrated. However, other important features such as ease of use, database functionality and traceability will also be discussed.

### Fast-automated characterization of polymers using pyrolysis gas chromatography/mass spectrometry

*Christopher R. Mubarak[1], Stephen L. Morgan[1] and Russell W. Zeigler[2], [1]University of South Carolina, Chemistry and Biochemistry, 631 Sumter Street, [1]212, Columbia, SC 29208-0001, USA,*

[2]*University of South Carolina, Chemistry, 631 Sumter St Csrc, Rm 212, Columbia, SC 29210, USA*

Achieving fast analysis of polymers remains an increasingly important task for many routine laboratories. Applications range from assessing stability to basic structural characterization. Analytical pyrolysis, in which gas chromatography (GC) or GC/mass spectrometry (MS) is coupled to a pyrolysis reactor, is a common approach for rapid polymer analysis. However, the GC separation itself is often the limiting step in decreasing overall analysis time. We have converted a HP-5890 Series II GCD quadrupole GC/MS into a high-speed separation instrument using a Thermo Orion EZ-Flash (TM). The column is resistively heated at temperature ramps as fast as 20 °C/s. Finally, by coupling a CDS Analytical Model 2500 Pyrolysis Autosampler to the GCD inlet, up to 45 samples can be analysed in an automated fashion. Analyses that conventionally required 30 min or more are now completed in 2–5 min, depending on the application. Because of heavy caseload requirements, forensic laboratories are continually looking for ways to increase sample throughput. As an example of fast polymer characterization for forensic analysis, we have investigated the possibility of automobile tire analysis. Rapid analysis of automobile tires could prove to be very useful for law-enforcement agencies in matching questioned evidence, such as tire residue, to a suspect tire. Even if such information cannot provide definitive identification, information from tire characterization may serve to reduce the number of possibilities in a forensic investigation. The combined EZ-Flash (TM)/pyrolysis autosampler/GCD instrument greatly increases sample throughput while producing data of sufficient quality to discriminate tires from different manufacturers.

### Automated plasma protein precipitation and filtration

*Sha Liao[1], Sarah Johnson[1], Susan Dawson[1] and Roger Roberts[2], [1]Hamilton Company, Instrument Marketing, 4970 Energy Way, Reno, NV 89502-4178, USA, [2]Ansys Technologies, Inc., 25200 Commercentre Drive, Lake Forest, CA 92630-8810, USA*

Biological sample preparation, particularly by removal of plasma protein, is an important step in drug metabolism and pharmacokinetic studies. Currently, most sample preparations are done manually using solid-phase extraction in low-density formats, or using a time-consuming centrifugation process. Automating plasma protein precipitation will dramatically increase the throughput of drug discovery. While some automated workstations have the capability of excellent liquid handling with air-displacement pipetting, the clogging tips caused by aspirating precipitated protein hampers their use in this application. Clogged wells have also been a problem for filtration of particulate material using the traditional 0.45-mm filter plate. In this presentation, a unique system combining positive displacement pipetting, disposable tips and vacuum, completely automates the process of preparing the particulate free, ready for use plasma in a 96-well format. This system uses a MICROLABa AT plus 2 automated robotic pipetting system and Captiva™ 96-well depth filter plates with a CaptiVac™ Vacuum Collar (Ansys, Lake Forest, CA, USA) to overcome dripping and clogging problems. This study presents a throughput comparison between the manual and automated processes, volume of filtration recovery and two standards recovery.

### Generic techniques in chiral method development

*Denise M. Wallworth and Thomas E. Beesley, Advanced Separation Technologies, Inc., 37 Leslie CT Box 297, Whippany, NJ 07981-1635, USA*

The process of using short, coupled columns for fast generic screening in chiral method development is examined. Rapid results in three different types of mobile phase systems are achieved enabling selection of a method according to the need. Molecules with a broad range of diverse stereogenic environments can demonstrate selectivity in < 2 h. Mobile phase design is critical to establishing the window of selectivity for very diverse racemates. This presentation will focus on the key issues involved in this design and present the rapid protocols for optimization of the final separation. Examples of screening several different molecular classes will also be given.

### Automated hit selection and consolidation

*Linda Aubin, Mike Dameron, Paul Lefebvre and Ray West, Gilson, Inc., Center for Integrated Discovery, 701 George Washington Highway, Lincoln, RI 02865, USA*

Much of the modern drug-discovery process is carried out in groups or sets of compounds, from compound synthesis to biological screening. The group format is often a microplate but may also be a set of tubes or vials. As compounds move through the process they are eliminated from consideration or advanced as 'leads'. The lead compounds are consolidated at various points in the process based on specified criteria and then moved on to the next step in the process. This process has been referred to as 'cherry-picking' or 'hit-picking' and is used to focus in on those compounds that are most likely to be viable drug candidates. Automated liquid handlers that can be used to consolidate compounds are important tools to improve the productivity of the process. For example, consolidation of synthetic compounds that pass quality control or positive hits from biological screening are applications. Three automated hit-picking systems will be compared here in terms of cost, throughput and capacity. The comparison will include system using disposable tips and fixed probes, single-probe and variable span, multiple-probe liquid handlers. The deck of the liquid handler is defined based on the source and destination plates or vials. Methodology is created to optimize such features as transfer amount, throughput and probe rinsing. Samples for consolidation are chosen graphically from the source plate layout or by importing a list from the user database. Upon completion, the user can generate a work list report.

**Automation of dynamic liquid-phase micro-extraction in determination of polycyclic aromatic hydrocarbon**

*Li Hou[1] and Hian Kee Lee[2], [1]Department of Chemistry, Lower Kent Ridge Road, 3 Scien, Singapore 119260, [2]Department of Chemistry & Biology, Provo, UT 84602, USA*

An automation of dynamic Liquid-Phase Micro-Extraction (LPME) technique combined with HPLC has been developed for the extraction of polycyclic aromatic hydrocarbon (PAHs) from water samples. In this study, the main objectives are: (1) automating the experiment with a Harvard (PHD2000) apparatus so that the experiment could be simplified; (2) obtain a large enrichment factor by increasing sampling volume and number of samplings; and (3) improve the precision. The factors influential to dynamic LPME were optimized with automation. The effects of organic solvent, plunger movement pattern, sampling volume, number of samplings, salt concentration and temperature were investigated. Analytically, the relative standard deviation is < 6%. Enrichment factors could reach over 280. By using a sampling volume of 20 μl and 40 samplings, the limits of detection were between 0.35 and 0.60 μg/l.

**Regulatory control and monitoring of contaminants and residues in Nigeria**

*Momodu S. Momodu, Dora N. Akunyili and S. Agegbu Denloye, Federal Secretariat Phase 11, Lagos, PMB 12949, Marina, Nigeria*

The control of persistent organic pollutants which are problematic in many developing countries has received adequate attention in Nigeria with the use of TLC, GC and HPLC techniques in NAFDAC laboratories. Residue data of organochlorines, organophosphorous and carbamate pesticides have been obtained. Impressive reports on the quality of formulations and quantities of residues from studies carried out so far indicate that capacity building in the field of contaminants and residues control is worthwhile. The routine evaluation of toxic metals in food, water and environmental samples using atomic absorption spectroscopy has helped in the setting of standards and national regulations. The scope of monitoring and control is being extended to mycotoxins; and veterinary drug residues with the prospect of use of ELISA kits. Data acquisition for regulatory control to promote consumer safety and international trade is expected from these studies.

**Long-term monitoring of nitrate in the Tangipahoa River**

*Jeremy S. Stevens[1] and John R. Allen[2], [1]Southeastern Louisiana University, Chemistry and Physics, SLU 878, Hammond, LA 70402-0001, USA, [2]Southeastern Louisiana University, 612 Oakhall Drive, Holly Springs, NC 27540-8719, USA*

The Tangipahoa River is an important river in the parish of Tangipahoa, Louisiana, USA. The river starts as far north as Liberty, Mississippi, and empties into Lake Ponchatrain. Unfortunately, the river has been prohibited to swimming and fishing due to environmental reasons. This research deals with the analysis of nitrate, which plays a role in the nitrogen cycle. There are many dairy farms that run along the river and sewage from homes which can be an important source of nitrate, a fertilizer for plants. At high concentrations nitrate is harmful to fish and man. Seven sampling sites from Tangipahoa Parish were chosen with six sites being highways that cross the river and the other by a local boat launch. Each sampling site was 3–5 miles from the next. Two representative samples were taken from each site along the river. The samples were then taken back to the laboratory for analysis by an ion-selective electrode for nitrate. This will give an indication of the fluctuations of nitrate over time. These fluctuations are compared with weather patterns during the time of testing. Under normal weather conditions, test results show that the concentration of nitrate decreases as it approaches Lake Ponchatrain. After a heavy rain, the concentration rapidly increases in areas that are normally low. This may be due to an increase of nitrate from the runoff from local dairy farms or sewer drainage.

**Characterization and evaluation of kaolin treated with ultrasonic energy and chemical agents in the hydrodesulfurization of a vacuum gas oil**

*Alfredo Castillo-Mares, Rebeca Silva-Rodrigo, Guillermo Sandoval-Robles, Aaron Melo-Banda and Ricardo García-Alamilla, Instituto Tecnológico de Ciudad Madero, Ingeniería Química y Bioquímica, Juventino Rosas y Jesus Urue, Cd Madero, Tamaulipas 89440, Mexico*

In the present work, kaolin was submitted to the action of chemical agents, such as HCl, $NH_4Cl$, $HNO_3$ and $NH_4Cl_2$M. solutions and treated with ultrasonic energy (US) or magnetic stirring and used as support of NiMo catalysts. Aluminium extracted was measured by atomic absorption while the acidity of the treated material was determined by the dehydration of isopropyl alcohol and potentiometric titration with *n*-butylamine. Surface properties were determined with an N2 physi-sorption and the structures by X-rays diffractograms. Hydrodesulfurization (HDS) was carried out in a batch reactor at 350C and 80 kg/cm$^2$ initial H2 pressure for 3 h. Sulfur removed was measured with an CHNOS analyser. Activation with chemical agents caused a dealumination being deeper when kaolin was acid treated than with the derived salt. The US effect reinforced this and dealumination values were close to 11 wt%. Total acidity from *n*-butylamine varied from 1.70 to 1.06 meq/g corresponding the highest values to the samples treated with US energy. Only propylene appeared in the chromatograms from the isopropyl alcohol dehydration indicating Bronsted type sites. The highest surface area was for the $HNO_3$ US treated sample being almost 10 higher than the raw material. Total pore volume varied from 0.051 to 0.139 cm$^3$/g and pore diameter from 208 to 46 Å. X-rays diffractograms showed a more amorphous material when US energy was used. US treated kaolin with HCl gave 49% HDS compared with a 14 and 34% with $HNO_3$ and a commercial catalyst, respectively.

### Quality control of a wheat gluten hydrolysate for use as a raw material in cell culture media

*Heather K. Schwartz[1], Matt Caple[1], Kevin Ray[1], Ben Cutak[1] and James Blasberg[2], [1]Sigma-Aldrich, 3300 S. 2nd Street Bldg N3, Saint Louis, MO 63118-3306, USA, [2]Sigma-Aldrich, Mallinckrodt & 2nd Streets, St Louis, MO 63147, USA*

Animal cell culture medium is a mixture of amino acids, vitamins and salts which is traditionally supplemented with serum or other animal-derived components. Protein hydrolysates prepared from a variety of sources including animal tissues, milk protein and plant tissue have been shown to be capable of replacing or reducing the need for more expensive components such as serum. More recently, due to increasing concern about the potential contamination of animal-derived components by adventitious agents, plant hydrolysates have been used to replace animal source medium components. In particular, we have found a wheat gluten hydrolysate, optimized for high levels of small peptides and rich in stable glutamine, to have particular value as a component in the formulation of serum-free cell culture medium. A decrease in productivity, with no corresponding decrease in cell growth, has been associated with lot-to-lot variability of this hydrolysate. We have developed an RP-HPLC method with photodiode array and MS detection for peptide mapping of wheat gluten hydrolysate in order to examine lot-to-lot variability. Significant differences noted in the profile are correlated to observed differences in the biological activity of cells grown in medium containing different lots of hydrolysate. The presentation will demonstrate an LC-PDA-MS method that can be used as a quality control method for wheat gluten hydrolysates.

### Search algorithms in an integrated analytical system for searching databases of spectra and chemical structures with improved searching results

*Michael Boruta and Marie Scandone, Bio-Rad Laboratories, Informatics Division, Sadtler Division, 3316 Spring Garden Street, Philadelphia, PA 19104-2552, USA*

All IR search systems for spectral interpretation and structure elucidation of organic compounds depend on the same principles. In them, a spectrum of an unknown compound is compared with all spectra in a reference database and the best matches are reported. Most systems assign a degree of similarity, which reflects the spectral properties common to the spectrum of the unknown compound and the reference compound. For all the reference compounds showing a degree of similarity, a chemical structure can usually be listed. This presentation will examine how a classification search can point to a class of compounds when an identification is not possible. Such a system can focus on the classification of the compounds listed and can control if an identity, a similarity or a classification search is performed to produce the best results. With this approach, it is possible to modify the search characteristics of a database search system in a predictable manner and improve searching results. Thus, a user can tune the system for maximum performance.

### Process control of pharmaceutical products using AOTF-NIR

*Igor Nazarov, Brimrose Corporation, Spectrometer Division, 5024 Campbell Blvd, Baltimore, MD 21236-5974, USA*

Our discussion will be on the development of our system to monitor and control processes of pharmaceutical products in stages from raw material ID to final product inspection. Our system uses various types of Brimrose AOTF-NIR Spectrometers, special operator's interfaces and software. Our system can be used to monitor production of solid dosage forms, liquids and powders in pharmaceutical and other industries. Advantages of AOTF-NIR spectrometers will be shown and demonstrated. We will present the Brimrose family of the highly reliable, fast (up to 16 000 wavelength $s^{-1}$) and on- and off-line analysers including the FreeSpace Spectrometer for non-contact diffuse reflectance measurements in 'open' ± space or through windows, 16 channels multiplexer for transmission, transflectance, diffuse reflectance measurements with different kind of fibreoptics. The new products including miniaturized Luminar 3075-701, battery operated, spectrometer that rides on pharmaceutical blenders and controls the uniformity in real time and the 'Universal' Luminar 3070 Tablet Analyzer that measure tablets on a moving belt in transmission and diffuse reflectance modes will be demonstrated. The Development of new 21 CFR Part 11-complied software to back it up for the pharmaceutical industry will be shown.

### Automatic HPLC method development: practical experiences with pharmaceutical and chemical samples

*Wolf-Dieter Beinert[1], Volker t Volker Eckert[1], Reinhold Spatz[1], Sergey Galushko[2], Irina Shishkina[3] and Vsevolod Tanchuck[3], [1]Merck KGaA, Scientific Laboratory Products, Frankfurterstrasse 250, Darmstadt D-64271, Germany, [2]Institute of Bioorganic Chemistry National Academy of Sciences, Fine Organic Synthese, Im Wiesengrund 49-b, Muehltal D-64367, Germany, [3]Institute of Bioorganic Chemistry of National Academy of Sciences, Murmanskaya 1, Kiev-94, Ukraine*

ChromSword Auto is an automated expert system developed to search for optimum conditions in reversed-phase HPLC. Parameters that can be optimized are concentration of an organic modifier for isocratic or gradient conditions and temperature. For proper peak tracking, ChromSword Auto offers two possibilities. In the most simple mode, a standard of each compound to be separated is required and the optimization procedure performed with the single standards only. However, often for all compounds to be separated suitable standards are not available. This is usually the case with pharmaceutical quality-control samples for purity and stability tests. The advanced peak tracking mode of ChromSword Auto allows one to perform the main part of the optimization process with the compound mixture itself and the number of standards necessary is reduced to only two, regardless of how many compounds are present in the mixture. As result of the optimization procedure, the samples are separated into a maximum number of peaks, with optimum resolution and minimum analysis time. By practical optimizations examples of real pharmaceutical and chemical samples, the possibilities of this new automated chromatography method development system are explained.

### Bioconnectors for discovery

*Scott Deutsch and Malcolm McGregor, LabVantage Solutions, 245 Highway 22, Bridgewater, NJ 08807-2560, USA*

Discovery science presents a severe challenge to companies attempting to make use of emerging techniques. To maintain competitive advantage, it is imperative that companies use cutting-edge technology as it becomes available. The result is a constantly changing environment that is difficult for coordinating and management systems to keep up with. A typical year-long purchase and implementation cycle that is common for laboratory management software becomes untenable under the circumstances found in discovery-focused organizations. Likewise, the inflexibility of these systems makes them sluggish, difficult and expensive to change. The individual nature of each laboratory due to the large number of options available for each subsystem, i.e. instruments, robotics, expert software systems as well as the differing requirements of the specific science, means that an out of the box solution is very difficult to implement. In fact, commercial packaged software vendors have found it difficult to carve a niche in this market. This paper explores a novel modular approach to providing the required commercial software with the tools required to meet the strictures outlined above. Attention is paid to providing small pieces that can be added and removed at will from the system. These are described in the system as BioConnectors™. Advantages include a rapidly implementable system that can be updated continuously by laboratory personnel as required. The paper also explores some of the challenges faced in keeping the modules up to date and transferable from operation to operation as well as maintaining the ability to support them commercially.

### Construction of LIMS systems using analytical data system

*Toshinobu Yanagisawa, Kiyoshi Yamashita, Yoshihiro Hayakawa, Atsushi Yoshida, Yasuhiro Funada and Teruhisa Ueda, Shimadzu, Analytical Instruments Division, 1 Nishinokyo-Kuwabaracho Nak, Kyoto 604-8511, Japan*

Construction of LIMS systems often requires developing custom-made software. However, many automation features such as sample sequence, data export and OLE automation have been implemented in an Analytical Data System. For example, more than 1000 samples can be analysed in a single HPLC system with an autosampler. Remote automation is easily achieved by using an OLE automation feature with Visual BASIC or other programming languages. The user interface can be customized for a specific purpose of application. One of problems in such a system is lack of integrity of data from different instruments such as HPLC, GC, LCMS, GCMS, UV, FT-IR and electric balance. It is not rare in a laboratory to analyse a sample with multiple instruments. Shimadzu LabSolutions Analytical Data Systems solved this issue by CLASS-Agent data management software using a back-end database on a network server. Oracle, MS-SQL server or MS-ACCESS can be used for the database. Raw data and calculation results from each instrument are stored to the database together with sample information such as sample name and sample ID. An application programme can be launched to perform custom calculations when each instrument stores data to the database. As data with the same sample information are easily queried, analysis results of the sample with multiple instruments can be browsed in the same platform. These results can be summarized to a single report. A generic interface to store data to the database is supported for multivendor instruments. These features contribute to enhance throughput in a laboratory. The focus of this presentation is to implement LIMS systems effectively by using current analytical data systems.

### The benefits of automation with a Laboratory Information Management System (LIMS) implemented at a chemical facility

*Mathew Abraham, Tom Miller and Danny Cormier, Accelerated Technology Laboratories, Inc., Engineering, 496 Holly Grove School Road, West End, NC 27376-8412, USA*

Chemical testing laboratories are under increased pressures to track a variety of samples in a rapid yet thorough way while complying with federal regulations and standards. Coastal Chemical Co. serves the natural gas processing markets as well as the refining, chemical and petrochemical markets. The analytical laboratory selected and implemented a Laboratory Information Management Systems (LIMS) to automate their login, enhance their data accessibility, incorporate a full chain of custody, implement a full audit trail, automatically track their sample status, provide QA/QC functionality, automate reporting and manage the vast array of laboratory data generated. This presentation will focus on the advantages of tracking samples, viewing sample status real time, viewing charted data, statistics with Sample Master Pro LIMS. The company required a data management system with great flexibility to track a myriad of samples and comply with federal regulations with integrated reporting. This presentation will focus on the LIMS selection criteria, implementation, and custom report creation. In addition, automation benefits realized following implementation will also be discussed.

### Laboratory Information Management System (LIMS) that spans the network and beyond

*Don Kolva, Tom Miller and Christine Paszko, Accelerated Technology Laboratories, Inc., 496 Holly Grove School Road, West End, NC 27376-8412, USA*

In the past few years, a laboratory that was implementing an LIMS had to decide whether it wanted a traditional client/server configuration or a browser-based LIMS. The client/server configuration is easier to modify and provides a better user interface, but the browser-based system allows for intra- and Internet access to users without the need to install any client side software. For many laboratories, the ideal solution is to implement one system that uses both interfaces. With a single LIMS that can use either configuration, the laboratory can choose the interface that is appropriate for the user. For example, an internal user can use a client/server configuration to take advantage of the speed, reliability and flexibility of an internal network. This configuration allows for better report modifications, data transfers to other applications such as Microsoft Office and a more traditional GUI interface (graphical user interface) for the user. Extending the same LIMS to an intra- or Internet allows remote users to view the information globally. It also provides a better interface for mobile users who may only be able to connect to the system via the Internet. A browser interface also uses software that is probably already on the computer, making maintenance easier than the client/server configuration. This presentation will explore the advantages and disadvantages of each configuration using real-life examples and will show that you can have your cake and eat it too!

### The role of users in an LIMS (Laboratory Information Management System) implementation

*Lisa Gorenflo, Jodie Roybal and Kim Paszko, Accelerated Technology Laboratories, Inc., 496 Holly Grove School Road, West End, NC 27376-8412, USA*

The human element is most often ignored during an LIMS project, but some LIMS project failures (or lack of full implementation) are due to human issues and lack of involvement by the users rather than technology issues. This presentation will look at ways of improving user involvement and hence increasing the probability of success for your laboratory's LIMS project. This presentation will help to answer the questions: How do you make an LIMS project successful and what are the reasons for failure? How do you involve users actively in your LIMS project? What are the styles of implementing your project involvement plan? How should you evaluate the types of users and match their involvement to phases of the LIMS project for best effect? Often different departments within a laboratory face unique challenges that other groups do not encounter. It is critical that the data management requirements of these groups are taken into account and with today's flexible LIMS system, special functions can be added and user's needs can be accommodated. Often times the Information Technology group has special requirements to match current IT infrastructure, in-house expertise, security requirements and future growth plans. It is critical that the various groups within the laboratory cooperate and participate in the selection process through the implementation. Those typically involved in the project team include; the laboratory manager, the IT manager, the business manager, QC group leader and there may be other departments. It is also helpful if one person can serve as the mediator, this person should understand the terminology and needs of all groups. A blueprint for a successful LIMS implementation with a focus on the human element will be described.

### ONQ, a system for automated quality assurance (QA) of on-line NIR analysers

*Kari Aaljoki, Automation Engineering, PO Box 310, Porvoo FIN-06101, Finland*

The success of on-line NIR-application depends on several factors that can be divided into three categories: sample handling (e.g. water and particle removal and temperature stability); analyser itself (e.g. repeatability and accuracy of spectra, and S/N); and modelling (e.g. coverage of calibration sample space, quality of input data, and skill). When the number of analysers and measurements increases, it becomes obvious that some sort of automated system is absolutely necessary for routine QA and continuous validation of the analyser farm, because doing these tasks manually would simply be too expensive. Without proper QA and validation NIR installations are bound to fail. The idea behind OnQ is simple. Spectra themselves contain a lot of information about above-mentioned categories. OnQ does rule based diagnosis of the spectra and SPC to pinpoint hardware or sample handling problems. It also uses SPC (Statistical Process Control) techniques to assess model performances based, e.g. prediction differences (lab-on-line result) and some multivariate measures of prediction statistics and the validity of the model used. It collects also data (spectra and reference values) at the same time for later model updates just to save time. OnQ features automated reporting, trending, and charting functions for major key variables for easy visual inspection. Problems are reported to maintenance people through e-mail. Technically speaking, OnQ has been built using Borland's Delphi 5 and it runs on Windows NT. OnQ uses MS SQL server as its database engine. Laboratory data from in-house-built Oracle 7.3-based LIMS running on HP9000 is collected using SQL queries. Some reference values from other on-line analysers are collected from ABB's PMS (Process Management System) using its ODBC capabilities. This paper shows some simple examples taken from real situations in order to demonstrate the usefulness automated QA and validation of NIR-analysers.

### On-line capillary electrophoresis instrument for industrial processes

*Stella Rovio[1], Teemu Työppönen[1], Pertti Vastamäki[1], Ari Hokkanen[1], Heli Sirén[1] and Pekka Savolahti[2], [1]Technical Research Centre of Finland, VTT, VTT Chemical Technology, Biologinkuja 7, Espoo FIN-02044, Finland, [2]Technical Research Centre of Finland, VTT, PO Box 1401, Espoo FIN-02044, Finland*

Capillary electrophoresis instrument for on-line control of industrial processes has been developed with continuous flow delivery systems for solvent and sample. The instrument consists of a three partial cell unit, the centre part for the capillary being changeable for fluorescence or UV detections and the front and rear modules with channels for solvent control. The on-line unit has fixed tunnels for dynamic replenishment of electrolyte solution at its both ends. Owing to the on-line solvent delivery system constructed into the cell unit, the capillary and the platinum electrodes will be kept uncontaminated. Samples are introduced into the capillary with pressure or electrokinetically or by using a combination of both the techniques. Samples with high ionic strength will be on-line diluted with an electrolyte solution before they are introduced into high-speed CE analyses. Voltages up to 2.5 kV can be used in separations. This presentation demonstrates the work done on the instrumentation and the advantages of dynamic sample and electrolyte feeding. Effect of flow conditions on pressure differences between capillary ends were studied by determining the electro-osmotic flows with different electrolyte feeding rates and by comparing migration times and peak shapes of separated sample peaks from the electropherogram. Electrophoretic separations with laser induced fluorescence detection (LIF) are demonstrated by using CAPS as background electrolyte for the analytes fluorescein, mandelic acid and phenyl acetic acid. Preliminary results for the samples oxalate, acetate and sulphate anion, detected by the UV detector at 254 nm wavelength were also performed by using OFM-PDC as a background electrolyte. On-line applications on pulp and paper process are demonstrated.

### Computer system validation and electronic records/signatures: a pharmaceutical laboratory perspective

*Sunday A. Brooks, Phyllis Hodson-Hutsell, Nancy Craig, Terry Tripp, Bryan K. Jones and Sandy Birge, Eli Lilly & Co., Tippecanoe Quality Control Labor, 1650 Lilly Road, Lafayette, IN 47909-9201, USA*

What does computer system validation (CSV) and electronic records/electronic signatures (ER/ES) mean to an analytical laboratory and its instrumentation? How is this process different from laboratory GMP qualification of an instrument? In response to 21 CFR Part 11 (Code of Federal Regulations), many laboratories are evaluating their laboratory instrumentation for compliance. This federal regulation also assumes that validation exists for computer systems. Until the passage of Part 11, many laboratory instruments were not validated as computer systems. This presentation will detail the planning and execution of the CSV plan for Eli Lilly Tippecanoe Quality Control Laboratory (QCL). Tippecanoe QCL and IT, guided by a Quality Control CSV Coordinator, have completed the analytical instruments' computer system validations. Laboratory CSV requires significant contributions from chemists or other technical experts familiar with each instrument. Laboratory personnel must define the instrument requirements and execute a testing plan which proves the capability of the instrument and its computer. Computer system validation of a laboratory instrument includes not only hardware/software review and testing, but also user training, system support and contingency planning. Action and implementation plans for ER/ES will also be discussed. Plans can include software upgrades, procedural controls, and physical security. Example plans for data integrity, security and archiving for specific types of instruments will be discussed.

### Quantification applications with sensor array systems: a new approach in quality control

*Quitterie Lucas*[1], *Vincent Schmitt*[2] *and Tsung Tan*[2], [1]*Alpha M.O.S., Application Laboratory, 3 Avenue Didier Daurat, Toulouse F-31400, France,* [2]*Alpha M.O.S.,* 20 Avenue Didier Daurat, Toulouse F-31400, France

The overall fragrance, flavour and aroma intensity perception is very important for the consumer. Achieving complete customer satisfaction and royalty requires that the perceived odour to be excellent quality and quantity all the time. These characteristics then ensure that luxury and premium brands maintain high profitability and market shares. The quality-control aspects ensure that the once the fragrance or aroma has been developed and demonstrated to meet the quality requirements of the consumer; in this presentation, we will focus on one case study of quantification: peppermint aroma in toothpaste. This industrial application will demonstrate the ability of the Sensor Array System for the quantification of the level of peppermint and it also demonstrates how such information can be used easily in the production environment to ensure constant quality and quantity while ensuring minimum use of expensive raw materials to achieve maximum results. The results obtained using calibration curve (PLS) and statistical Quality Control (SQC) data for the quantification and control of peppermint quantity in toothpaste demonstrated the ability of FOX4000 and GEMINI Sensor Array Systems for use in many industries where a high level of QC/QA is required to ensure satisfied and loyal customers.

### Automation of dilutions for various sample types: liquids, solids and difficult samples

*Lynn Jordan, Jayne Brown and Stephanie Wilson, Zymark Corporation, Zymark Ctr, 68 Elm Street, Hopkinton, MA 01748-1668, USA*

One of the most common operations in any laboratory is dilutions. Two common uses of dilutions are to prepare standards for calibration of analytical equipment, and to put samples into solution. In many laboratories, preparing standards and samples is a daily operation. Dilution of standards or samples is most commonly done manually using volumetric flasks. This method of performing a dilution yields an accurate result, but involves a manual operation, offers no documentation unless it is performed on a balance, and in many instances generates more sample or standard than is necessary for the application. This poster will explore the use of an automated workstation to perform dilutions for multiple types of samples, and provide 21 CFR Part 11 documentation. Examples will be shown for dilutions of solid samples, liquid samples as well as 'difficult samples' such as suspensions or creams. The manual method will be compared with an automated method, and the following parameters will be investigated: operator time, overall time to prepare samples and waste generated documentation of the dilution.

**Possibilities for automation of sample preparation steps before LC or GC analysis using a common autosampler**

*Eike Kleine-Benne[1], Friedhelm Rogies[1], Peter Popp[2] and Andrea Buhr[3], [1]Gerstel GmbH & Co. KG, R&D, Aktienstrasse 232–232, Muelheim An Der Ruhr D-45473, Germany, [2]UFZ—Umweltforschungszentrum Leipzig-Halle GmbH, Sektion Analytik, Permoserstra E 15, Leipzig D-04301, Germany, [3]Fraunhofer Institut für Holzforschung, Wilhelm-Klauditz-Institut, Bienroder Weg 54e, Braunschweig D-38108, Germany*

Primarily autosamplers for chromatographic devices are used to increase the degree of utilization. A second important reason to use autosamplers is the improved reproducibility and comparability of analytical results because of their independence from different users. This is accepted for sample injection and is important for sample preparation. A source of possible errors for sample preparation steps is the operator. A software solution is presented how a common autosampler for LC or GC (MPS, GERSTEL GmbH & Co. KG, Germany) can be used to avoid such errors without purchasing expensive laboratory robots. A simple example is presented how to prepare standard solutions for daily calibration. A more complicated example shows a complete sample derivatization (esterification) followed by solvent extraction (toluene) and transfer of the extract in autosampler vials. A further application shows how the system in combination with a membrane extraction inlet for headspace vials is used for liquid–liquid extraction of chlorinated pesticides in aqueous samples with hexane. After extraction the same autosampler is used to inject volumes of up to 100 μl of the extract for GC analysis without further clean up. In combination with a flow through cuvette and a LC injection valve an on-line coupling of a LC and a GC system is possible whereas the autosampler is used for injection into the LC, fraction collection from the cuvette and injection into the GC. A special application is the fully automated extraction of the GERSTEL Twister with acetonitrile–water mixtures followed by injection into an LC for analysis of PAH in aqueous samples.

**Comparison of automated headspace techniques static headspace, multiple headspace extraction, dilution and purge-and-trap using gas chromatography**

*Ingo Christ, LEAP Technologies, PO Box 969, Carrboro, NC 27510-0969, USA*

Static headspace methods are widely used for volatile and semivolatile compounds. They are environmentally friendly because they involve a solventless procedure. Many different techniques have been developed to eliminate the matrix effects of headspace methods, to simplify them or to increase their sensitivity, reproducibility and the speed of their analyses. Examples of these techniques include Purge-and-Trap, Multiple Headspace Extraction (MHE) and a simple dilution series designed to create a calibration curve. Autosamplers can automate most of the steps required to perform these techniques; however, the fact that autosamplers typically are limited to performing one specific technique is problematic in that it is difficult to know which technique might work best for each sample. Additionally, it is not easy to compare the results of the analyses due to the difficulty of transforming from one technique to another. This difficulty is primarily caused by differences in sample handling and physical variances in sample containers. This article will compare the results of each technique and discuss practical applications. Since a unified sample container will be used, comparison among the different techniques will be possible. Using a standard sample in a standard headspace vial, the results of a static headspace analysis will be compared with a Purge-and-Trap method and with MHE and sample dilution. All four methods will be software-controlled and performed without changing hardware. Sample preparation will be automated to eliminate manual error. Linearity and reproducibility of the automated methods will be shown to compare favourably with manual ones.

**An automated approach to the removal of plasma protein precipitates by filtration**

*Hung Nguyen, William Hudson, Roger Roberts and Dennis D. Blevins, Ansys Technologies, Inc., 25200 Commercentre Drive, Lake Forest, CA 92630-8810, USA*

A new 96-well filtration product is evaluated on two automated liquid-handling platforms for the purpose of performing Plasma Protein Precipitation sample preparation. The Captiva (TM) 96-well filter plate is an all-polypropylene depth filter with a minimum pore size of 0.45 μm that is quality controlled for cleanliness of materials and non-specific binding of analytes during filtration of plasma protein precipitates. The precipitating reagent and plasma samples were mixed and introduced into the Captiva 96-well filter plate by the automated platforms. The fast-flowing filter design prevents clogging. The precipitate-free samples were collected and submitted for analysis by HPLC. In this paper, two automated protocols for plasma protein precipitation filtration and supporting HPLC analytical data for the recovery of tricyclic antidepressants and a comparison to typical plasma protein precipitation by centrifugation are presented. The data demonstrates a clean, fast, and effective automated method of performing plasma protein precipitation filtration.

**Development of a multipurpose device (Auto-Shot sampler) for automated introduction of samples into the microfurnace type pyrolysers dedicated to pyrolysis-gas chromatography (PY-GC)**

*Chuichi Watanabe[1], Yoshio Kawahara[1], Kunitaka Sato[2], Paul Tobias[3], Hajime Ohtani[4] and Shin Tsuge[4], [1]Frontier Laboratories Ltd, R&D, 1-8-14 Saikon, Koriyama, Fukushima 963-8862, Japan, [2]Frontier Laboratories Ltd, R&D, 61-2 Otsubo, Koriyama, Fukushima 963-0201, Japan, [3]Quantum Analytics, Inc., 363 Vintage Park Drive, Foster City, CA 94404-1135, USA, [4]Nagoya University, Department of Applied Chemistry, Chikusa-Ku, Furo-Cho, Nagoya, Aichi 464-8603, Japan*

Automated sample introduction for PY-GC is playing a very important role in the analysis operations especially in the quality or process control of polymeric materials.

The automation of the measuring system greatly enhances the efficiency of analysis by saving labour and minimizes analytical errors caused by human manipulation. In the last Pittsburgh Conference, we have presented a device, Auto-Shot Sampler AS-1020E, directly attached to a microfurnace type pyrolyser of PY-2020iD (Frontier Lab, Japan). This automated sample introduction device was only applicable to the flash pyrolysis of up to 48 samples at a given pyrolysis temperature. This paper reports another capability added to the previous features since then. The new feature is that the sample cup can be moved up and down vertically between the centre of the microfurnace of the pyrolyser and the waiting position in the Auto-Shot sampler by the push of the pressurized carrier gas. With this capability, two analytical functions, stepwise analysis (combination of thermal desorption and pyrolysis), and heart-cutting GC analysis (correcting and analysing any desired portion of evolved gas from the sample during temperature programming) became effective. This entire operation can be automatically controlled independently for each of the 48 samples mounted in a sample tray by specially configured software installed in a personal computer.

### An automated 96-well one-does-all extraction method for drugs of abuse in oral fluid

*William C. Hudson[1], Dennis D. Blevins[1] and David O. Hall[2], [1]Ansys Technologies, Inc., 25200 Commercentre Drive, Lake Forest, CA 92630-8810, USA, [2]Ansys Technologies, Inc., 605 Vernon Tharp Street, Columbus, OH 43210-4007, USA*

Automating sample preparation provides an efficient means to free staff from mundane tasks, which are prone to processing errors. With modern robotic systems, high-throughput laboratories can dedicate expensive equipment to perform a single analysis in a repetitive fashion. The key advantages include sample throughput and traceability. With large robotic systems, personnel are dedicated to software development and systems integration, providing the support to make the automation requirements cost effective. The combination of a 'one does all' extraction on an automated platform facilitates the practicality of a small laboratory investing in an automated system. In the past, different drug types have required different extraction protocols. On an automated system, each drug type would require a unique extraction programme and set of solvents. Combining different drug types into one extraction protocol allows a laboratory to simplify all extraction protocols (saving time) and standardize the use of solvents and reagents (saving money). The confirmation of drugs of abuse in oral fluid presents several challenges to clinical/toxicological laboratory analysis. The lack of available sample, compared with urine, and meeting the cut-off levels drafted by SAMSHA, increase the difficulty in performing the confirmation of multiple drugs in one sample. By developing an extraction protocol of 12 major drugs of abuse (amphetamine, methamphetamine, MDA, MDMA, MDEA, PCP, cocaine, benzoylecgonine, THC, codeine, morphine, and 6-monoacetylmorpine), it becomes possible to report multiple drugs from one analysis and simplify multiple laboratory protocols into one analysis. The sample was extracted using an ANSYS Technologies SPEC-Ora-Lab-96-Well plate in a Hamilton AT 2 Plus robotic system. The Hamilton robot performs all sample pretreatments, transfers and washes on an automated platform. The sample and wash volumes used were adapted to the Hamilton's low volume pipettes to minimize sample aspiration and dispensing. Samples were subsequently concentrated, derivatized by MSTFA and analysed by GCMS. Samples were evaluated by extraction recoveries (most absolute recoveries were approximately 80%), and precision and accuracy studies were run over 3 days period. This presentation will summarize the automated protocol and describe laboratory timesavings versus manual extraction protocols.

### Using an automated vacuum system for 96-well filtration and extraction

*Susan C. Dawson, Sarah Johnson and Sha Liao, Hamilton Co., 4970 Energy Way, Reno, NV 89502-4178, USA*

The key to high-throughput solid-phase extraction (SPE) and filtration applications is automation of the 96-well microplate format. The Hamilton Co. MICROLAB Automated Vacuum System integrates a motorized vacuum manifold and a vacuum controller into an automated liquid-handling workstation. A variety of commonly available 96-well filter plates and collection plates are used to prepare biological and other samples via SPE or filtration. The vacuum box is motorized so that the filter plate can be transported from a waste chamber to an elution chamber. The manifold is compact, and two can be used in parallel on the deck of the pipetting workstation. Filter plates can be placed onto or removed from the system manually or with a robotic arm. The vacuum controller uses feedback monitoring to maintain the vacuum at the user-defined set point. In this presentation, applications and analytical results of filtration and extraction using this vacuum system will be described.

### Automated acid reflux cleaning method for Teflon, glass and quartz accessories used in trace analysis

*Robert Richter, Milestone, Inc., Department of Chemistry and Biochemistry, 160B Shelton Road, Monroe, CT 06468-2545, USA*

Analytical chemists take numerous precautions to ensure the lowest possible analytical blank. These precautions include the use of high-purity acids and reagents for sample preparation, powder-free gloves, lint-free protective clothing, rigorous cleaning of all sample preparation and instrument components, etc. Despite all of these precautions, many chemists still have problems controlling the analytical blank. Their problems usually results from the way the sample preparation and instrument components (i.e. Teflon bottles, volumetric flasks, glass and quartz ICP/ICP-MS spray chambers and torches, sample and solution containers, microwave vessels, etc.) are cleaned and stored. Milestone has developed the TraceCLEAN system to address these problems. The TraceCLEAN system is unique in that is uses sub-boiling distillation to clean various sample preparation and instrument components. Sub-boiling distillation is the same technique used to prepare high-purity acids used for analysis. This means components are not contaminated by the acid used to clean them. Another important feature of the TraceCLEAN system is that the cleaning and drying of the components occurs in a sealed container. This minimizes the amount of airborne contamination that can be deposited on the components, keeping them clean until they are needed. This poster compares the cleaning of TFM microwave digestion vessels and high-purity quartz inserts using the TraceCLEAN, with the traditional method of cleaning these components.

### Interstage monitoring: the only way to guarantee the purity of ultra-pure water

*Paul Whitehead and Alan Mortimer, ELGA, R&D, High Street, Lane End, High Wycombe HP14 3JH, UK*

All ultra-pure water systems include a de-ionization purification step based on ion-exchange resin. This poster will describe the major benefits to be gained by splitting the de-ionization system into two stages and monitoring the water purity interstage. Interstage resistivity monitoring enables the first ion exchange cartridge to be replaced when its exchange capacity is exhausted while the second stage is virtually unused. This ensures that all water passes through fully regenerated ion exchange resin before dispense, guaranteeing the water purity. This approach eliminates dependence on accurate product water resistivity monitoring, which is known to be difficult and costly, and the reliance on frequent checks on the outlet water quality. Ion-exchange resin utilization is maximized, the cost of cartridge exchange is reduced and the release into the product water of weakly bound components, such as organic compounds, silica and boron, are avoided. Dividing the de-ionization into two stages also enables the optimum location of the ultraviolet chamber interstage and permits the use of cost effective integral TOC monitoring.

### Speciation of mercury using atomic fluorescence spectrometry

*Derek W. Bryce, Warren T. Corns and Peter B. Stockwell, PS Analytical Ltd, Arthur House Crayfields Industrial Estate, Main Road, Orpington BR5 3HP, UK*

It is common knowledge that the toxicity of mercury is extremely dependant on the form in which it is present. In particular, organic mercury species are for more toxic than the inorganic forms. There is a great need to be able to fully speciate mercury in order to gain further knowledge of its toxicity transformation, transportation and bioavailability. The two most frequently taken approaches to mercury speciation involve either HPLC or GC separation followed by element specific atomic spectrometric detection. Atomic fluorescence spectrometry is an ideal detector for use in such systems as it shows unrivalled sensitivity, linearity, stability and freedom from interferences. It is also extremely easy to couple to either HPLC or GC systems, and is economical to run and maintain. The paper will describe two approaches for the speciation of mercury using atomic fluorescence detection. The first system, using reverse phase HPLC-atomic fluorescence spectrometry allows the speciation of mercury in aqueous samples without the need for any derivitization or extraction into organic solvent. On elution from the column, UV oxidation is used to convert organic mercury to $Hg^{2+}$ before reduction to $Hg^0$ with $SnCl_2$. A preconcentration step may also be employed to reach lower levels. The second approach uses GC separation of mercury species using a non-polar megabore capillary DB-1 column. As the species elute from the column, they pass through a pyrolysis unit to convert the mercury to $Hg^0$ before it enters the AFS detector. Advantages and disadvantages of each system will be reviewed and extraction procedures for a range of sample types, presented, along with results for a range of samples and certified reference materials.

### Atomic fluorescence: 15 years' experience of the technique as a measurement tool for mercury in laboratory and process stream applications

*Peter B. Stockwell, Warren T. Corns, Derek W. Bryce and Jasmina Allen, PS Analytical Ltd, Arthur House Crayfields Industrial Estate, Main Road, Orpington BR5 3HP, UK*

Over the past 15 years there has been considerable regulatory activity relating to the determination of mercury in both laboratory and process applications. PS Analytical and its academic and industrial partners have been actively involved in developing the chemical procedures required to measure accurately and reliably the levels of mercury in many matrices. Procedures are available for all European and US (EPA) regulatory needs for including EPA 1631, 245.7 and CEN PrEN 13506. Procedures involving continuous flow analysis, discrete sample analysis and amalgamation coupled to these procedures will be described with particular reference to the matrices for which each is particularly useful. The sensitivity, linearity and ruggedness of the technique also makes it an ideal approach for process monitoring applications. However, these various applications provide extremely difficult analytical applications. Several approaches to solving particular issues relating to incinerator waste, chlor-alkali and sulfuric acid matrices will be described in detail.

### On-line continuous flow liquid membrane extraction-C18 precolumn-based trace enrichment for the determination of sulfonylurea herbicides in water by liquid chromatography

*Gui-Bin Jiang[1,2], Jing-Fu Liu[1] and Jing-Bo Chao[1], [1]Research Centre for Eco-Environment, PO Box 2871, Beijing 100085, P. R. China, [2]Faculdade de Ciencias Farmaceutc, Braganca Paulista 12900-000, Brazil*

Coupling on-line a continuous flow liquid membrane extraction (CFLME) device and a short C18 precolumn with HPLC, a novel automatic system was developed for the trace determination of sulfonylurea herbicides in water. After preconcentration by CFLME, which is the combination of continuous flow liquid–liquid extraction and support liquid membrane (SLM) extraction, the target analytes were enriched in 950 μl of 0.5 M $Na_2CO_3$-$NaHCO_3$ (pH 10.0) buffer used as acceptor. This acceptor was neutralized and transported to a C18 precolumn (30 × 4.6 mm) where analytes were absorbed and focused. Then the focused analytes were injected onto a C18 analytical column for separation and detected at 240 nm with a diode array detector. Metsulfuron methyl (MSM), sulfometuron methyl (SMM), and DPX-A 7881 were separated with a mobile phase consists of methanol and acetic acid–sodium acetic buffer at a flow rate of 1 ml $min^{-1}$. With an enrichment time of 40 min and enrichment sample volume of 80 ml, the enrichment factors for these three sulfonylurea herbicides are about 1000. This proposed method was applied to determine sulfonylurea herbicides in river water, sea-water, lake water, tap water, bottled mineral water. Compared with the existing SLM-short C18 precol-umn-HPLC method, this proposed procedure has the advantages of higher system stability, higher enrichment factor with shorter analytical time, and lower detection limits.

### Automation of multidimensional chromatography for protein purification in high-throughput structural studies

*Thomas Pless, Pär Eklund and Jill A. Sigrell, Amersham Pharmacia Biotech, R&D, Björkgatan 30, Uppsala SE-751 84, Sweden*

Structural genomics, also known as structural proteomics, has taken off. To determine structures, by both X-ray crystallography and NMR, fairly large amounts (> 10 mg) of target protein in a pure and homogenous form are usually required. Therefore, simple high-throughput purification of proteins is needed by structural biologists. The flexible ÄKTA platform can be configured in different ways to suit preferred purification schemes. By using a new configuration on ÄKTAexplorer, supplemented with two seven-port valves and two sample loops, automated two-step purifications of different proteins can be performed serially. Soluble, well expressed, proteins tagged with either (His)6 or glutathione *S*-transferase (GST) were used in the evaluation of this new configuration. Two different purification schemes were compared: (1) affinity chromatography followed by gel filtration and (2) affinity chromatography followed by an intermediate desalting step and ion exchange chromatography. Both methods have the capacity to produce up to 50 mg of 90–99% pure target protein. With this new configuration, the researcher saves time and reduces protein losses compared with the same chromatography steps performed manually.

### Large-scale protein identification using automated gel processing and high-throughput MALDI mass spectrometry

*James Langridge[1], Jeff Brown[1], Ronan O'Malley[1], Alistair Wallace[1], Dominic Gostick[1] and Philip Young[2], [1]Micromass UK Ltd, Life Sciences, Floats Road, Manchester M23 9LZ, UK, [2]Micromass UK Ltd, Department of Chemistry, PO Box 904, Emory, VA 24327-0904, USA*

The huge increase in genomic sequence information available has allowed the large scale, high-throughput identification of proteins (usually separated by 2D PAGE) by mass spectrometry, Matrix assisted laser desorption ionization (MALDI) time of flight mass spectrometry provides an ideal method for protein identification usually with fmole levels of sample where the acquisition and processing of the data can be easily automated. The provision of samples for high-throughput automated MALDI TOF mass spectrometry has been addressed by the introduction of robotics. By using a gel-cutting robot, excision of single or multiple 1 mm diameter gel cores into a 96-well microtitre plate well can be easily achieved. The software image of the gel is then annotated with the excised spot number and tracks the sample position in the microtitre plate. The microtitre plate can be transferred to an automated in-gel digestion robot which performs reduction, alkylation, in-gel tryptic digestion and peptide extraction for the gel samples followed by automated spotting of the peptides onto a MALDI target plate. This robot is totally enclosed to prevent external contamination of the gel samples. In this paper we describe a bench-top MALDI mass spectrometer fitted with an automatic plate-changing robot. All data acquisition, processing and searching is performed in an automated mode (unattended operation). The resulting protein identifications can then be directly linked with the gel image. This approach will be discussed, with specific examples obtained from the large scale analysis of samples originating from 2D gel electrophoresis.

### DNA hybridization detection at ultratrace level by flow injection surface plasmon resonance

*Fayi Song, Jianqiao Lin and Feimeng Zhou, California State University, Chemistry and Biochemistry, 5151 State University Drive, Los Angeles, CA 90032-4226, USA*

A flow-injection device is combined, through the use of a low-volume thin-layer flow cell, with a sensitive surface plasmon resonance (SPR) spectrometer equipped with a biocell photodiode detector. The preliminary results showed that the resultant flow injection SPR (FI-SPR) device could be applied for high-throughput, sequence-specific DNA analyses at ultracetrace levels. Self-assembled monolayers (SAMs) of a 30-base oligonucleotide whose 3′ end is derivatized with a mercaptohexyl tether group (HS-ss-ODN) were immobilized onto the thin Au film and then used as a model heterogeneous sensor for the analysis of a 47mer target. The DNA hybridization assay based on this FI-SPR device was also found to possess a high sample throughput and excellent sample specificity. The sensitivity of the FI-SPR will be compared with that of other types of SPR and several ultrasensitive techniques for labelled DNA targets (i.e. fluorescence-tagged and radiolabelled DNA samples). The use of a hexylamine-terminated 30mer probe cross-linked onto a preformed SAM of thiolated succinimidylundecanoate will be tried to further decrease the detection level. The possibility of using SPR for detection of a small number of ODN molecules will be exploited. An application of detecting *Arabidopsis thaliana* mRNA expression pattern during transcription process will be attempted using this system.

### Development of continuous monitoring system for gas generation behaviour during coking reaction

*Masayuki Nishifuji, Koji Saito and Yuji Fujioka, Nippon Steel Corporation, Advanced Technology Research Lab, 20-1 Shintomi, Futtsu, Chiba 293-8511, Japan*

To characterize coking reaction of coal in iron-making process, it can be important information to realize gas generation behaviour on pyrolysis of coal. In this study, a new monitoring system for gas generation behaviour during coking reaction has been established. This system consists of three parts, a carrier and atmosphere gas supply line, a reaction tube (heating a sample with an electric furnace) and two detectors of Fourier-transform infrared spectrophotometer (FT-IR) and of a sensor for hydrogen which cannot be detected by FT-IR. Consequently, this system enables one to monitor all generated gases simultaneously. Coal powder sample in nitrogen gas flow was heated, and generated gases were directly lead to detectors with nitrogen gas carrier. This method has a good time-resolution of roughly 10 s with an improved cell without staying of gas flow, so that a short time reaction of coal pyrolysis can be monitored. Using this system, different gas profiles of two typical coals during coking reaction, which have quite different characters in iron-making process, could be obtained. This result well agreed with a differential curve of sample gravity obtained by the thermogravimetric analysis. It was revealed that this system has a great reliability of quantity. Furthermore, the present system is great effective in the characterization of coal pyrolysis, because it is able to monitor continuously the reaction *in situ* up to high temperature (> 600 °C) other methods cannot examine.

### Using robotics to automate beryllium analysis by inductively coupled plasma-atomic emission spectroscopy

*Gerald L. DeVault, Greg G. Howard, Craig C. Hanzelka, Ed L. Churnetski and E. Ray Hinton, The Y-12 National Security Complex, Analytical Chemistry, 113C Union Valley Road, Oak Ridge, TN 37830-8045, USA*

A robot has been developed to automate the sample preparation of filter media for beryllium (Be) analysis by inductively coupled plasma-atomic emission spectroscopy (ICP-AES). The robot increases the sample throughput while reducing the potential exposure to analytical technicians. Beryllium exposure can result in a lung disease known as beryllіosis, consequently workplace contamination must be continuously monitored. Beryllium has been used extensively in the nuclear industry and can be found as a solid, a powder, or in a compound such as beryllium oxide. Typically, filter media is used for both air and surface monitoring, followed by labour intensive sample dissolution and analysis by ICP-AES. In contrast, the robot automates the sample preparation and interfaces with the ICP-AES instrument resulting in complete automation of the analysis. The filter samples are placed in 50-ml plastic cones labelled with a bar code. The robot transports the sample between stations where a number of functions are performed, such as reading a barcode, filter dissolution, addition of an internal standard, and dilution. After the sample preparation is complete, the robot signals the ICP-AES instrument to start sample analysis. In 24 h, about 300 samples can be prepared, analysed and reported. Filters ($n = 40$) spiked with 0.20 μg Be-nitrate were analysed and a mean of 0.20 μg (±0.01 μg) was obtained. A detection limit of 0.10 μg of Be/filter has been demonstrated. Implementation of the ICP-AES robot will increase the efficiency of the laboratory without reducing the quality of the analytical results.

### Flow-injection system for on-line olive oil emulsification and inductively coupled plasma-mass spectrometry multi-elemental determinations and *in-situ* emulsions stabilization for direct determination of iron and chromium in olive oil by electrothermal AT

*Juan R. Castillo, Maria S. Jimenez and Rosario Velarte, University of Zaragoza, Analytical Chemistry, Ciudad Universitaria Scienca, Zaragoza, Spain*

In this study we propose first the on-line preparation of emulsions by FI for multi-elemental determination in olive oil by ICP-MS. Besides another analytical interest of this study is to check the possibility of using aqueous calibration solutions to quantify olive oil samples and characterize different types of olive oils from different Spanish areas Optimization of the chemical conditions for the on-line formation and stabilization of olive oil emulsions will be reported: type of emulsifier, emulsifier concentration, sample amount, pH, ultrasonic stirring time, salinity. In addition, optimization of FI variables will be reported: amount of olive oil sample, flow rates, lengths and diameters of the different tubing segments and ultrasonic stirring reactor. As a complementary work we propose the determination of Fe and Cr in different types of olive oil samples by GFAAS with direct oil emulsion formation by adding directly the sample and the emulsifier in water solution to the autosampler cup. The emulsion is formed by ultrasonic agitation with a high intensity ultrasonic processor used normally for slurries formation. Once the emulsion is formed, the autosampler injects the olive oil emulsion in the graphite furnace and Fe and Cr are determined by AAS. This method is very low time consuming and the sample pretreatment takes only 10 s. Different parameters regarding to the emulsion formation have been optimized: sample (olive oil) amount, emulsifier concentration, time of agitation, ultrasonic power of agitation. In addition, parameters to do with the GFAAS determination have been studied: temperature programme and the use of modifiers.

Castillo, J. R., Jimenez, M. S. and Ebdon, L. *Journal of Analytical and Atomic Spectrometry*, **14** (1999), 1515.

### New on-line trace boron analyser for ultrapure water

*Richard D. Godec[1], Paul Kosenka[1] and Karla Dennis[2], [1]Ionics Instruments, Inc., Sievers New Product Development, 6060 Spine Road, Boulder, CO 80301-3323, USA, [2]Intel, Corporate Services Facility Tech, 2501 NW 229th Avenue, Hillsboro, OR 97124-5503, USA*

We have developed a sensitive on-line analyser to measure trace levels of boron in ultrapure water. This analyser is used in the semiconductor industry to control the levels of boron in the ultrapure water used to make semiconductors. The analytical method will be described. The data from a performance study at an Intel semiconductor factory will be reviewed. The study compared recovery of boron standards from 0.030 to 2.0 ppb as boron in ultrapure water using high-resolution ICP-MS and the new on-line Boron Analyzer. The results indicate the boron standards' analysis data from the on-line Boron Analyzer and the high-resolution ICP-MS are equivalent.

### A screening method for polycyclic aromatic hydrocarbons using hollow fibre membrane solvent micro-extraction

*Stephanie King, Jill Meyer and Anthony R. J. Andrews, Ohio University, Chemistry and Biochemistry, 171 Clippenger Labs, Athens, OH 45701, USA*

As regulatory agencies are faced with increasing workloads, the development of a fast, low cost, reduced waste method to screen samples is crucial. A rising environmental concern is the contamination of water and soil with polycyclic aromatic hydrocarbons (PAHs). Long-term exposure to PAHs has resulted in cataracts, kidney and liver damage, reproductive difficulties, and many different types of cancer. Sources of PAH contamination include leakage from storage tank linings and burning of oil and fossil fuels. Solvent Micro-Extraction (SME) is an effective method for analyte preconcentration providing low limits of detection. However, in viscous solutions such as soil, where rapid stirring improves extraction efficiency, SME is often hindered by drop dislodgement. For this study we used hollow fibre membrane solvent micro-extraction (HFMSME). A total of 8 $\mu$l of extraction solvent was injected into a hollow, porous fibre that was sealed on one end.

### Automated on-line monitoring of VOC from the PAMS list

*Philippe Verdeguer[1,2] and Franck Amiet[1], [1]Chromato-Sud, R&D, 15 Route D'Artiguelongue, F-33240 Saint-Antoine, France, [2]Chromato-Sud, Department of Chemistry, 179 Chemistry Bldg, Auburn, AL 36849-5312, USA*

Volatile organic compounds (VOC) represent more than 300 identified compounds. Non-methane hydrocarbons from two to 10 carbon atom number are the main part of VOC in urban ambient air. Owing to their negative impact on human health, they are now considered are first priority pollutants. Benzene and 1,3-butadiene are known to be highly carcinogenic. Moreover, they have been recognized to play, together with nitrogen oxides, an active part in tropospheric ozone formation. For these reasons, governmental authorities now recommend the survey of individual VOC. In Europe, experts agreed on a list of 30 compounds, while in the USA 50 compounds have been retained in the famous PAMS list. The airmOzone rack offers the possibility of monitoring continuously a wide range of ozone precursors down to ppt levels. The system is constituted of two gas chromatographs (4 U), with each unit being dedicated to the analysis of a specific range of compounds: the AirmoVOC C2–C5 allows the analysis of C2 to C5 VOCs, as the AirmoVOC C6–C10 is dedicated to higher compounds. The two chromatographs can be integrated in a cabinet equipped with an $H_2$ generator, a zero air generator and a permeation unit making a complete automatic and stand alone system with no need of external cylinder. Data on the stability of the system in terms of retention time and peak areas as well as the linearity of the response will be shown. The capabilities of the software as well as the possibility to performing remote control and calibration of the system will be demonstrated. Finally, real life examples with identification of VOCs from the PAMS list will be presented. The sample is stirred at 1350 rpm. After the optimized 8-min extraction time, 4 $\mu$l of the solvent was withdrawn and directly injected for analysis. Using an HP 6890 gas chromatograph with flame ionization detection, 17 PAHs were identified based on retention time and further quantified. Detection limits were calculated based on a signal-to-noise ratio of 3 and range from 0.132 to 0.22 mg kg$^{-1}$ in soil. PAHs with low molecular weights gave lower limits of detection because of their increased solubility in water. Those with higher molecular weights were not extracted as efficiently. Calculated extraction efficiencies in soil varied from 1.8 to 8.2%. HFMSME provides a quick, simple, low cost screening method for environmental samples. Because the 8-min extraction can occur during the 10-min GC run, five samples can be analysed per hour. Each extraction used one fibre costing less than 1 pence and very small volumes of organic solvent. No extensive filtering or pretreatment of the sample was necessary and the limited equipment required would allow this technique to be used for on-site analysis.

### An automated solid-phase micro-extraction of ethyl carbamate in wines using gas chromatography/mass spectrometry

*Eshwar Jagerdeo[1] and Ingo Christ[2], [1]Department of Treasury, Bureau of Alcohol, Tobacco and Firearms, Department of Treasury, 1401 Research Blvd, Rockville, MD* 20850-3159,

USA, [2]*Leap Technologies, PO Box 969, Carrboro, NC 27510-0969, USA*

Ethyl carbamate (EC), also known as urethane, is a known carcinogen found in various food and alcohol beverages. In fermented alcohol beverages, it is formed as a natural by-product. Currently, the US Food and Drug Administration (FDA) is doing the toxicological evaluation of EC with an aim of establishing maximum tolerance levels of this contaminant in food and beverages. Various methods have been published for the determination of EC in food and beverages. These methods involve an extensive sample preparation procedure with the potential for matrix interferences. This article discusses a method for the analysis of EC in wines using an automated solid-phase extraction (SPME) and a gas chromatography coupled to a mass spectrometer in the chemical ionization mode. In this method, various conditions and parameters of the fibres and sample preparation are optimized for the quantitation of EC in wines. The use of a dual-arm autosampler helps in the automation of the sample preparation. Furthermore, the SPME procedure uses a heated magnet-mixing chamber to reduce the sample extraction time. This method eliminates the need for extensive sample preparation and affords EC determination and confirmation in the low ppb. This procedure is quick, and affords excellent quantitation and reproducibility.

### Automated on-line monitoring of volatile sulfur compounds for environmental and industrial applications

*Philippe Verdeguer*[1,2] *and Franck Amiet*[1], [1]*Chromato-Sud, R&D, 15 Route D'Artiguelongue, F-33240 Saint-Antoine, France,* [2]*Chromato-Sud, Department of Chemistry, 179 Chemistry Bldg, Auburn, AL 36849-5312, USA*

Because of their impacts on environment and human health, the analysis of volatile sulfur compounds has become a necessity. This paper presents some applications of the new generation of Airmomedor analyser. Based on gas chromatography with a specific electrochemical detector for sulfur compounds, this on-line analyser allows the survey of $H_2S$, COS, mercaptans and sulfides at the ppb level. It can be integrated in a cabinet with a zero air generator, a permeation unit making a complete stand-alone unit with no need of external cylinder. It can operate in a fully automatic mode including remote control capabilities and automatic validation. Datas on stability and linearity of the response will be shown. Different applications will be presented including fermentation process control, quality of $CO_2$ or other gas process, waste water plant treatment process control, continuous emission monitoring and air monitoring stations.

### The combination of an automated trapping system with a portable GC

*Wolf Muenchmeyer and Andreas Walte, WMA Airsense, Hagenower Str. 73, Schwerin, MV D-19061, Germany*

A new trapping system was developed in order to combine it with a portable GC. The detection system consists of a micro-GC using a thermal conductivity detector. For some applications the detection limit of about 1–10 ppm for the detector is too high. Sometimes there is also a higher selectivity needed. With an external enrichment unit, it is possible to lower the detection limit by one or two dimensions, depending on the sampling time and the retention capabilities of the adsorptive material. By choosing an optimal adsorptive material with the corresponding sampling temperature, a selective enrichment is also possible. Disturbing solvent peaks or other main peaks due to humidity can be eliminated or minimized. After sampling, the adsorption tubes are thermally desorbed and analysed. Applications showing the reproducibility and the improved detection limits will be shown.

### Improvement of sample carryover in the autosampler of HPLC

*Nobuyuki Tatsumi, Yoshiaki Maeda, Tsuyoshi Morikawa, Yoshiaki Aso, Shuzo Maruyama and Teruhisa Ueda, Shimadzu Corporation, 1 Nishinokyo-Kuwabaracho Nak, Kyoto 604-8511, Japan*

Sample carryover becomes one of the most important performance to evaluate the HPLC system especially for those who uses HPLC in high-sensitivity analysis. As the MSMS detection is getting popular in the high-sensitivity HPLC analysis such as pharmacokinetic analysis, it appears that the carryover in the HPLC system is sometimes too much to be ignored because the sensitivity of the MSMS is much higher compared with the UV-VIS detector and the more the sensitivity is, the higher the carryover is observed. There are several causes of the carryover and it depends on the chemical characteristics of samples, flow line configuration of the autosampler, washing liquid and procedure of the autosampler, etc. However the two major cause of carry over are a ionic interaction between the sample and the metallic parts of a flow line such as a sample-sucking needle and a hydrophobic interaction between sample and polymeric material in a flow line such as Vespel or PEEK used for a switching valve of the autosampler or other plumbing. In this study, several types of sample-sucking needle have been compared when concerned with the carryover performance with both ionic and non-ionic substances. In addition, some materials used for the plumbing such as SS or PEEK tubing have been examined for chemical interactions to eliminate the carryover of HPLC system.

### Liquid chromatography/mass spectrometry with on-line photochemical derivatization of selected indole compounds

*Beata A. Musial, Abdulqawi Numan and Neil Danielson, Miami University, Chemistry and Biochemistry, 112 Hughes Hall, Oxford, OH 45056, USA*

Over the years, mass spectrometry (MS) has been proven to be the method of choice for obtaining important molecular ion information of many compounds. The high sensitivity and reliability of MS have attracted many scientists from various fields. The identification of isomers and closely related compounds is an existing problem with mass spectrometry especially if tandem mass spectrometry (MS/MS) is not available. Previously, we found that the combination of on-line photoderivatization and flow injection analysis-mass spectrometry (FIA/MS) can be successfully applied for the identification of aromatic compounds such as sulfonamides that have very similar structures. A photoreactor with the dimension of 2.5 m × 0.25 nm and a volume of 0.12 ml was used. Without photolysis sulfonamides tend to follow a common fragmentation pathway. Upon photolysis, however, each sulfa drug showed a unique mass spectrum that makes its identification much simpler. In the present work, the on-line photochemistry and MS of indoles will be addressed. Indole derivatives such as serotonin, tryptamin, tryptophan, melatonin and indole-3-acetic acid exhibit a UV absorbance in the region 250–290 nm, which makes their identification with UV-Vis difficult. The photolysis of indoles is of interest since some can be connected to the photodegradation of protein and some are of interest as antioxidants. For this work, Teflon tubing reactor (4 m × 0.1 mm i.d., 0.4 mm o.d.) will be tested. We have found that the volume of the photoreactor has a major role on the degree of the phototransformation of the indole compounds. Additionally, the nature of the mobile phase as well as the flow rates influence the photolysis products of the indoles. Finally, the LC separation of a mixture of indoles with on-line photolysis and MS will be addressed.

